# Biocontrol agents transform the stability and functional characteristics of the grape phyllosphere microenvironment

**DOI:** 10.3389/fpls.2024.1439776

**Published:** 2024-10-16

**Authors:** Tao He, Meng Yang, Hongyan Du, Ronghui Du, Yueqiu He, Sheng Wang, Weiping Deng, Yixiang Liu, Xiahong He, Youyong Zhu, Shusheng Zhu, Fei Du

**Affiliations:** ^1^ State Key Laboratory for Conservation and Utilization of Bio-Resources in Yunnan, Yunnan Agricultural University, Kunming, China; ^2^ Key Laboratory for Agro-Biodiversity and Pest Control of Ministry of Education, Yunnan Agricultural University, Kunming, China; ^3^ Institute of Ecological Agriculture in Hot Areas, Yunnan Academy of Agricultural Sciences, Yuanmou, Yunnan, China; ^4^ Key Laboratory for Forest Resources Conservation and Utilization in the Southwest Mountains of China, Ministry of Education, Southwest Forestry University, Kunming, China

**Keywords:** grape, powdery mildew, phyllosphere microorganism, *B. subtilis*, microorganism network, functional characteristics

## Abstract

The spread of grape leaf diseases has a negative impact on the sustainable development of agriculture. Diseases induced by *Uncinula necator* significantly affect the quality of grapes. *Bacillus* biocontrol agents have been proven effective in disease management. However, limited research has been conducted on the impact of biocontrol agents on the assembly and potential functions of plant phyllosphere microbial communities. This study used high-throughput sequencing combined with bioinformatics analysis and culture omics technology for analysis. The results showed that biocontrol bacteria *B. subtilis* utilized in this study can significantly reduce the disease index of powdery mildew (*p*<0.05); concurrently, it exhibits a lower disease index compared to traditional fungicides. A comprehensive analysis has revealed that biocontrol bacteria have no significant impact on the diversity of phyllosphere fungi and bacteria, while fungicides can significantly reduce bacterial diversity. Additionally, biocontrol agents can increase the complexity of fungal networks and enhance the degree of modularity and stability of the bacterial network. The results also showed that the biocontrol agents, which contained a high amount of *B. subtilis*, were able to effectively colonize the grapevine phyllosphere, creating a microenvironment that significantly inhibits pathogenic bacteria on grape leaves while enhancing leaf photosynthetic capacity. In conclusion, biocontrol agents significantly reduce the grape powdery mildew disease index, promote a microenvironment conducive to symbiotic microorganisms and beneficial bacteria, and enhance plant photosynthetic capacity. These findings provide a basis for promoting biocontrol agents and offer valuable insights into sustainable agriculture development.

## Graphical Abstract

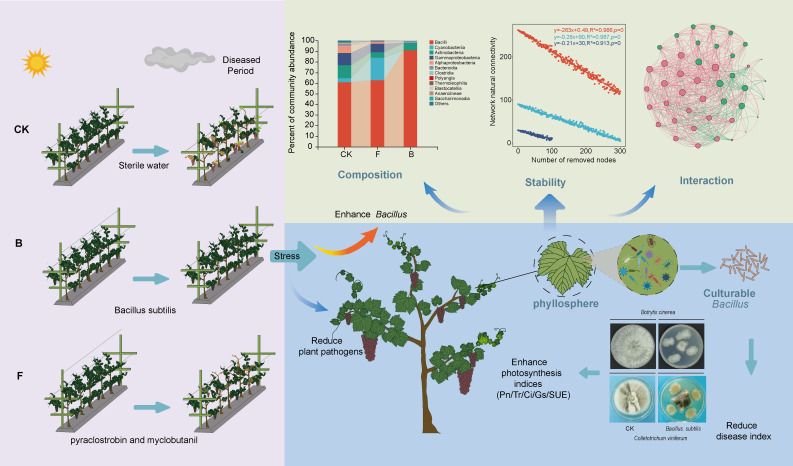

## 1 Introduction

Commercial table grape planting is burgeoning in China, particularly with the widespread implementation of rain-shelter cultivation to combat epidemic diseases, such as grape downy mildew and anthracnose (Du et al., 2015). As the demand for high-quality products drives the expansion of vineyards, the area dedicated to grape cultivation has also increased. This growth has contributed to the resurgence of a longstanding disease: grape powdery mildew, which is caused by *Uncinula necator* (Schw.) Burr. The onset of powdery mildew infection is marked by symptoms such as yellowing, leaf drop, and stunted growth of grape leaves (Warneke et al., 2020) posing a susceptibility challenge to the majority of cultivated grape varieties. As a result, growers are compelled to apply fungicides in substantial amounts, leading to a significant surge in production costs and a notable impact on human health and the environment (Jones et al., 2014; Nesler et al., 2015). As the area of rain-shelter cultivation expands in China, the incidence of powdery mildew across various production regions is increasing, leading to substantial losses in grape production (Calonnec et al., 2004). Presently, grape powdery mildew is primarily managed through chemical control methods, with commonly used fungicides such as Myclobutanil and Pyraclostrobin. Myclobutanil, a highly effective triazole fungicide with low toxicity, primarily targets the inhibition of ergosterol biosynthesis in pathogenic fungi, providing broad-spectrum control (Li et al., 2023). While pyraclostrobin is a methoxyacrylate fungicide that can induce physiological changes in various plants, enhancing nitroreductase activity and nitrogen uptake during rapid growth stages. Furthermore, both can stimulate the synthesis of endogenous hormones such as auxin and cytokinin (Hou et al., 2022; Li et al., 2020b). However, the use of broad-spectrum fungicides often leads to unintended consequences, impacting non-targeted microorganisms. These effects can ultimately disturb the microecological balance of plants. Studies have shown that fungicide usage can reduce bacterial community abundance in leaf tissue at the family and genus levels, particularly affecting bacteria involved in plant growth and nutrient supplementation (Ke et al., 2021).

The phyllosphere encompasses the aerial portions of plants and harbors a diverse array of bacteria, fungi, and other microorganisms. Establishing a resilient microbial community structure on the phyllosphere is essential for sustaining optimal growth conditions for host plants and enhancing crop disease resistance (Li et al., 2021b). Certain beneficial microorganisms associated with plant defense mechanisms can inhabit grape leaf margins, outcompeting pathogens for nutrients and interaction sites (Saleem, 2021). These microorganisms exert inhibitory effects through antimicrobial compounds, interfere with pathogen release signals, or induce resistance in plants (Li et al., 2021a).

*B. subtilis* employs both direct and indirect mechanisms for biological control, effectively inhibiting diseases caused by pathogens. This bacterium secretes secondary metabolites that disrupt the cell structures of pathogenic bacteria, induce plant resistance, and enhance the enzymatic activity associated with plant disease resistance (Tahir et al., 2017; Hashem et al., 2019). *B. subtilis* is widely utilized for the management of grape powdery mildew, exhibiting remarkable effectiveness. Kanitkar et al. (2020) reported that *B. subtilis* KTSB 1015 1.5 A.S. can be combined with triazole systemic fungicides to achieve a control rate of 99.98% on leaf powdery mildew and 92.31% on grape berries. Notably, this treatment resulted in a substantial increase of 63.32% in grape output. The composition and network stability of the plant phyllosphere microbial community can be influenced by various fungicides (Xiang et al., 2022). Following the application of Validamycin, a substantial increase on the diversity of phyllosphere fungi was observed in asymptomatic plant leaves. Moreover, the abundance and diversity of bacteria in both asymptomatic and symptomatic leaves exhibited an initial increase, followed by a subsequent decrease (Guo et al., 2024). For instance, the application of iprodione significantly alters microbial community structures both in plant phyllospheres and rhizospheres (Katsoula et al., 2020). Nevertheless, the effects of frequently employed fungicides within vineyards on grape phyllosphere microorganisms remains inadequately elucidated.

There is a significant gap in research regarding the impact of various agents used to control grape powdery mildew on grape phyllosphere microorganisms. Our objectives include: 1) Evaluate the efficacy of different agents (pyraclostrobin + myclobutanil, *B. subtilis*) in the treatment with grape powdery mildew; 2) Probing the influence of these agents on the composition and diversity of grape phyllosphere microbial communities, with an added aim of predicting the functional traits and roles of these microorganisms;3) Investigation the stability of the phyllosphere microbial network on grape leaves under different agents. Through these endeavors, we aim to establish a comprehensive theoretical framework to guide the development of antifungal agents within the realm of plant pathology.

## 2 Materials and methods

### 2.1 Site description

The study was conducted from July to September 2020 in the vineyards of the modern research and Teaching Practice Center, affiliated with Yunnan Agricultural University. These vineyards are located in Xundian County, Kunming City, Yunnan Province, China, positioned at 103°40’ E longitude and 25°23’ N latitude, with an elevation of 1920 meters above sea level. The research material selected for this study consisted of 5-year-old Red Globe grapes in the fruiting stage. The experimental site covered an area of 3 acres and under a rain-shelter system to mitigate the impacts of the local rainy season, which typically spans from July to September. This period is characterized by an average rainfall of 100 mm and a mean temperature of 25°C. Despite the observation of sporadic powdery mildew outbreaks in the previous year (2019), no fungicidal treatments were applied. The 2020 trial was initiated during the occurrence of powdery mildew in the rain-sheltered cultivation area.

### 2.2 Experimental design

This investigation focused on evaluating the effectiveness of 3,000-fold diluted concentrations of 40% myclobutanil and 3000-fold diluted concentrations of 25% pyraclostrobin (provided by Jiangsu Yunnong Chemical Co., Ltd) alongside a *B. subtilis-*OCASB12 (JQ240640.1) microbial foliar fertilizer, which has a concentration of ≥1×10^^9^ CFU/mL (supplied by Yunnan Province Microbial Fermentation Engineering Research Center Co., Ltd). The experimental design comprised three distinct treatments: (1) a combination of fungicides (3000-fold diluted pyraclostrobin and myclobutanil, with concentration of 8.6×10^^3^ μg/mL and 1.15×10^^3^ μg/mL, referred to as ‘F’); (2) a biocontrol treatment using *B. subtilis* (diluted 100-fold to achieve 1×10^^7^ CFU/mL, referred to as ‘B’); and (3) a control group that received applications of sterilized water (denoted as ‘CK’). For the application of biocontrol agents, the microbial concentration was drawn into a medical-grade sterile syringe under a sterile workbench environment, mixed with sterile water to prepare a suspension at a concentration of 1×10^^7^ CFU/mL, and subsequently transferred to a sterile small spray bottle for application. In the field, the treatments were applied separately to the front and back of grape leaves across three different groups. Each treatment plot measured 10 meters by 10 meters, with three replicates per treatment, resulting in a total of six plots. Treatment applications were conducted at seven-day intervals using a small-scale sprayer, culminating in a total of four applications.

Disease assessment involved the random selection of six vines per treatment prior to each application. From each vine, ten leaves were sampled to evaluate the extent of disease manifestation, adhering to a predefined leaf disease classification standard. Disease severity was graded on a scale from 0 to 9, Level 0: no disease; Level 1: the area of ​​lesions is less than 1% to 5% of the total leaf area; Level 3: the area of ​​lesions accounts for 6% to 25% of the total leaf area; Level 5: the area of ​​lesions accounts for 26% to 50% of the total leaf area; Level 7: the area of ​​lesions accounts for 50% to 75% of the total leaf area; Level 9: the area of ​​lesions is greater than 75% of the total leaf area, and the disease index is calculated according to the formula (Dong et al., 2023).


$$\begin{array}{c} \text{Disease index}=\sum \left(\text{Number of diseased leaves at all levels}\right)\\ \;\;\;\;\;\times \text{Representative value at all levels})\\ \;/\left(\text{total leaves investigated×Highest representative value}\right)\times 100 \end{array}$$

### 2.3 Sample collection

Sample collection was conducted 24 hours after the fourth and final field application. In accordance with the principle of homogeneous sampling, foliage that was free from precipitation, such as rain or dew, was meticulously selected. For each treatment group, three grapevines with comparable growth vigor, spaced 1.5 meters apart, were chosen. The sampling protocol focused on leaves that faced the sunrise and were maintained at a consistent height from the ground. Six replicates were collected for each treatment, with each sample carefully labeled and preserved in a 50 mL sterile centrifuge tube. The samples were immediately placed in a dry ice container for transport to the laboratory. Each gram of leaf material was treated with 10 mL of phosphate-buffered saline (PBS) solution (0.1 mol/L, pH 8.0). The samples underwent sonication for 15 minutes at 40 kHz, followed by a 10-second vortex at 20°C and 200 rpm. This washing procedure was repeated to ensure the thorough removal of pathogens. The two wash eluates from each sample were combined in a centrifuge tube and centrifuged at 4°C and 13,000 rpm for 10 minutes. The supernatant was discarded, and the resulting pellet was collected for subsequent analysis.

### 2.4 Total DNA extraction and PCR amplification of grape phyllospheric microorganisms

The precipitated samples were processed for DNA extraction using the Mp Fast DNA^®^ Kit (6560-200) from Omega Bio-tek, Norcross, GA, USA. The integrity and purity of the extracted DNA were verified through 1% agarose gel electrophoresis, followed by quantification with a Nano Drop 2000 spectrophotometer. Primers 338F (5’-ACTCCTACGGGAGGCAGCAG-3’) and 806R (5’-GGACTACHVGGGTWTCTAAT-3’) to amplify the V3-V4 region of 16S rRNA for bacteria, primers ITS1F (5’-CTTGGTCATTTAGAGGAAGTAA-3’) and ITS2R (5’-GCTGCGTTCTTCATCGATGC-3’) ITS1 region for fungi (Chen et al., 2022; Wen et al., 2020). Primers were synthesized by Shanghai Meiji Biomedical Technology Co., Ltd. Polymerase chain reactions were composed of a 20 µL solution, including reagents in optimal concentrations for the highest fidelity of amplification. The protocol for the reaction was set to initiate with a denaturation step at 95°C,: 95°C pre-denaturation for 3 min, 30 cycles (95°C denaturation for 30 s, 55°C annealing for 30 s, 72°C extension for 45 s), 72°C stable extension for 10 min, 10°C storage, 20 μL amplification system: 4 μL 5×Fast Pfu buffer, 0.4 μL TransStart^®^ FastPfu DNA Polymerase, 2 μL dNTPs (2.5 mmol/L), 0.8 μL upstream primer (5 μmol/L), 0.8 μL downstream primer (5 μmol/L), 1 μL DNA template (10 ng/μL), and ddH_2_O was added to make up to 20 μL. After amplification, the DNA amplicon was subjected to a purification step to ensure the removal of any non-target sequences. PCR products were extracted from 2% agarose gels and purified with the AxyPrep DNA Gel Extraction Kit (Axygen Biosciences, Union City, CA, USA) according to the manufacturer’s instructions, then quantified using Quantus (Promega, USA). The purified amplicons were pooled for sequencing on the Illumina MiSeq PE300 platform, with sequencing services provided by Shanghai Meiji Biotechnology Co., Ltd. The raw reads were deposited in the NCBI Sequence Read Archive (SRA) database (PRJNA1111966, PRJNA1112077).

### 2.5 Isolation of culturable microorganisms and evaluation of *B. subtilis* for pathogen control and photosynthetic performance in grapes

Cultivable microorganisms from the phyllosphere were isolated using the precipitate obtained from sample pretreatment. A portion of the precipitate was utilized to isolate and purify the interstitial microorganisms with B treatment, employing potato dextrose agar (PDA) as the culture medium. *B. subtilis* was isolated and subjected to a confrontation assay against the primary grape pathogens, including *Colletotrichum viniferum* GZ026 and *Botrytis cinerea* CLM13704 (Yunnan Agricultural University Crop Diversity Control Disease Laboratory Strain Storage Bank). *B. subtilis* was spot inoculated around each pathogen. The culture plates were then incubated in the dark at 28°C for 4 days. After the incubation period, the diameter of the inhibition zone around the pathogen disc was measured (in millimeters) with a caliper and recorded. Three replicates were set for each treatment, for a total of two treatments.

Grapes grown in a growth chamber were selected for research on photosynthesis. During the shoot growth period, 24 plants with similar growth were chosen, with 12 plants designated as the control group (CK), and the remaining 12 plants as the *B. subtilis* (B) treatment group (*Bacillus*). The B group was evenly sprayed with a 100-times diluted *B. subtilis* suspension six times, and their photosynthetic indexes were measured the following day. The GFS-3000, a photosynthesis measuring instrument from Germany, was used to measure the net photosynthetic rate (Pn), transpiration rate (Tr), stomatal conductance (Gs), intercellular CO_2_, concentration (Ci), and instantaneous light energy use efficiency (SUE), water use efficiency (WUE), photosynthetically active radiation (PAR), instantaneous carboxylation efficiency (CUE). The test was conducted under the following conditions: a light intensity of 1200 lx, a temperature of 28°C, and an indoor CO_2_ concentration.

### 2.6 Data analysis

The quality of the original sequence data was controlled using Fastp software (Chen et al., 2018), while Flash software was utilized for splicing, and Uparse software was employed to perform Operational taxonomic unit (OTU) clustering on the sequences and eliminate chimeras at a 97% similarity level (Stackebrandt and Goebel, 1994). The RDP classifier algorithm was used to classify and annotate the species of each sequence, with a comparison threshold of 70%, and compared with the 16SrRNA database Silva and ITS database Unite (Wang et al., 2007). Duncan’s new multiple range method was used to determine the significance of field experiments. The ANOSIM test was used in combination with Nonmetric multidimensional scaling (NMDS) and PCoA to analyze the beta diversity of fungi and bacteria. The differences at the microbial genus level were analyzed using Lefse (Fuentes et al., 2020), with a threshold of 4. The T-test was employed to analyze the differences in the complexity of the collinear network, SIMPER analysis and Spearman analysis were completed on the Shanghai Major Biotechnology Co., Ltd(https://www.majorbio.com/web/www/index). The Hiplot platform (https://hiplot.com.cn/home/index.html), Gephi 7.0 software (https://gephi.org/), GraphPad 8.0 (https://www.graphpad.com/), and Adobe Illustrator 25.2 (https://www.adobe.com/products/illustrator/campaign/pricing.html) were utilized for visualization and analysis.

## 3 Results

### 3.1 Efficacy of different treatments on grape powdery mildew

**Figure 1:**
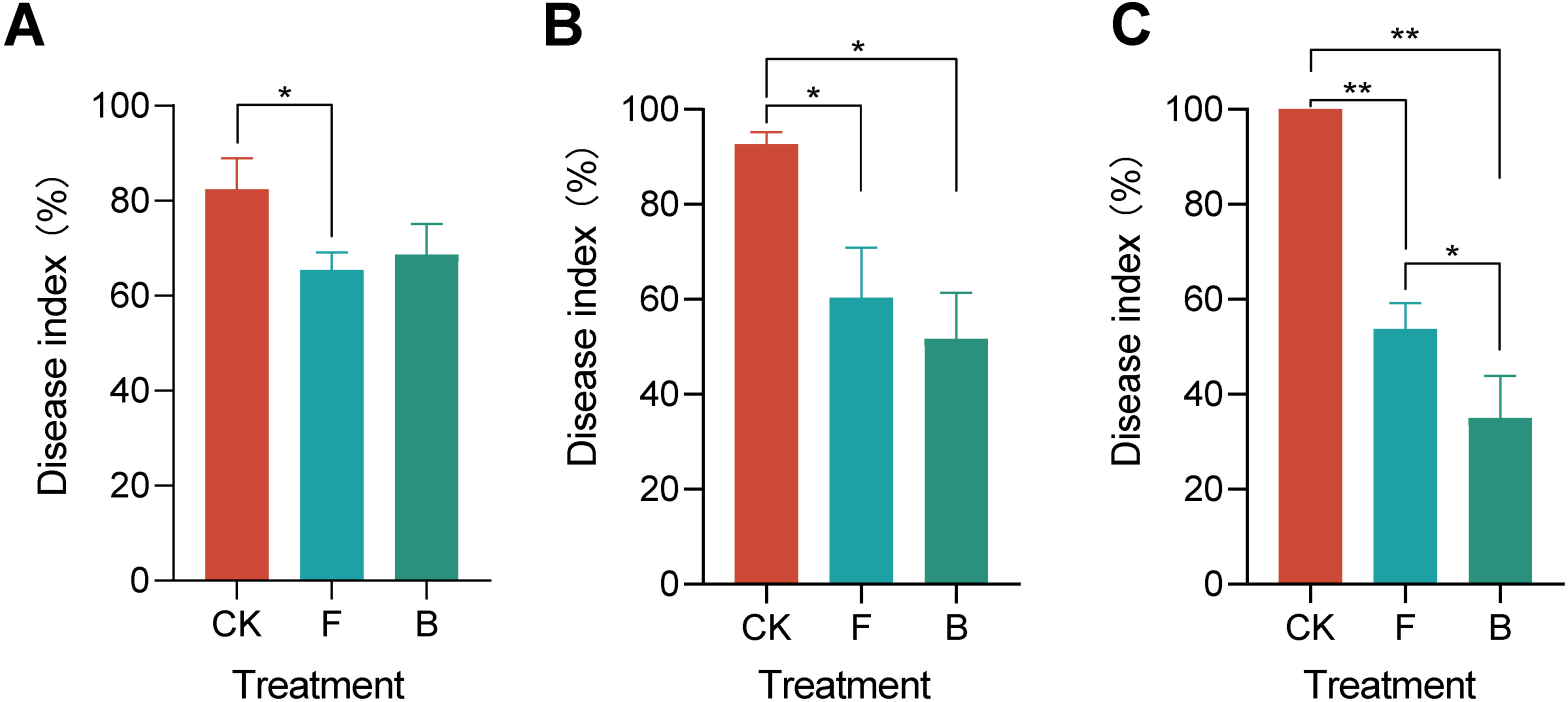
Grapevine powdery mildew disease index under different treatments. **(A)** Indicates the disease index of grape powdery mildew 2 days after treatment; **(B)** Disease index after 7 days of treatment; **(C)** Disease index of grape powdery mildew after 14 days of treatment. “*” indicates *p <*0.05, “**” *p <*0.01.

The results demonstrated that after 2 days, treatment F led to a significant reduction in the disease index of grape powdery mildew (*p*<0.05), while treatment B did not show a significant difference (**Figure 1A**). A subsequent evaluation at 7 days revealed that both treatments F and B significantly reduced the disease index of powdery mildew (**Figure 1B**). At the end of the 14-day trial period Both treatments F and B resulted in the powdery mildew index of grapes reducing to below 60%, which is significantly lower than that observed in the control treatment (CK) (*p*<0.01). Furthermore, the powdery mildew index for treatment B decreased even further to 35% (**Figure 1C**). The disease index of grape powdery mildew prior to the initiation of the experiment is presented in **Supplementary Figure 1A**. These findings indicate that treatments F and B effectively suppressed powdery mildew on grapes, demonstrating sustained efficacy. Based on these results, we hypothesize that the composition of the microbial community on the phyllosphere region of grapevines may be influenced by different pesticide applications, consequently impacting the incidence of powdery mildew.

### 3.2 Effects of different treatments on grapevine phyllosphere microbiota

#### 3.2.1 Alpha and beta diversity

**Figure 2:**
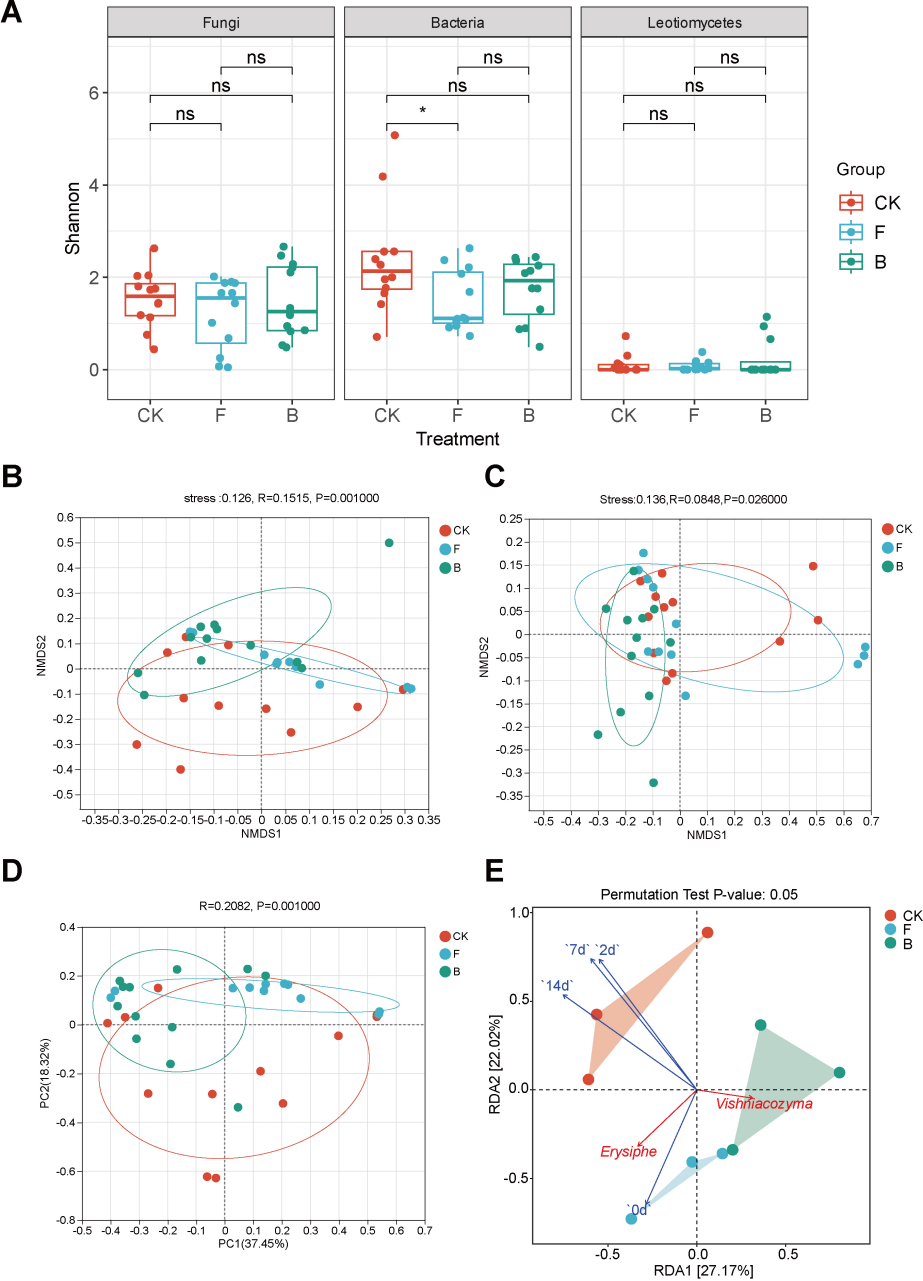
Analysis of microbial diversity on grapevine phyllosphere with powdery mildew disease. **(A)** Shannon index box plot on grape phyllosphere fungi, bacteria and Leotiomycetes; **(B)** NMDS analysis at the OTU level of fungal communities on grape leaves, depicted with various colors denoting distinct groups. The proximity of two treatment groups on the chart reflects the similarity in the structure of their respective microbial communities. The horizontal and vertical axes indicate relative distance, while the Stress value denotes the reliability of the NMDS analysis. A Stress value below 0.2 implies that the analysis offers strong explanatory power; **(C)** NMDS analysis at the OTU level within grape leaf bacterial communities; **(D)** PCoA analysis at the genus level of Leotiomycetes, with the axes representing the principal components and the percentages indicating the variance explained in sample composition; **(E)** RDA analysis correlating the powdery mildew disease index with prevalent fungi, against the background of the fungal community. “ns” indicates no significant difference after Student’s T test. (p >0.05), “*” indicates p <0.05.

Sample sequencing is reliable and captures most species. (**Supplementary Figures 1B, C**). The analysis of *α*-diversity on grape phyllosphere microorganisms at the OTU level demonstrated that following the application of different treatments, the shannon diversity of grape leaf bacteria was significantly reduced after treatment with fungicide (F) (*p* < 0.05) (**Figure 2A**), but it not reach a significant level after treatment with biocontrol agent (B). The remaining indices are shown in **Table 1**. Given that grape powdery mildew is caused by Leotiomycetes at the class level, we continued to evaluate the diversity of microorganisms in this group and determined that the Shannon index of Leotiomycetes was not significantly different among the three treatments (**Figure 2A**).

**Table 1:** Fungal and bacterial diversity index table under different treatments.

Microorganism	Treatment	Chao1 richness index	Ace richness index	Simpson diversity index
Fungi	CK	290.81 ± 186.18a	290.89 ± 185.87a	0.24 ± 0.26a
	F	224.80 ± 121.20ab	234.04 ± 114.76ab	0.61 ± 0.25ab
	B	121.81 ± 30.84b	140.80 ± 39.40b	0.43 ± 0.28ab
Bacteria	CK	32.92 ± 17.80c	31.32 ± 21.51c	0.44 ± 0.31ab
	F	14.83 ± 7.65c	12.37 ± 10.76c	0.76 ± 0.26b
	B	53.00 ± 62.46c	46.47 ± 67.47c	0.42 ± 0.30ab

The results presented in the table are expressed as “mean ± standard.” Different letters indicate significant differences (p < 0.05) based on the Student’s t-test.

An analysis of beta diversity was conducted to elucidate the impacts of various treatments on the microorganisms within the grape phyllosphere. The NMDS analysis was conducted at the OTU level utilizing the Bray-Curtis distance algorithm. The analysis of fungi revealed that, grapevine samples following F and B treatments were predominantly distributed across distinct quadrants, diverging from the CK group (**Figure 2B**). Stress values below 0.1 suggested an effective NMDS model configuration. Subsequent ANOSIM analysis, employing a nonparametric test, resulted in an *R* value of 0.1515 and a *p*-value less than 0.01, denoting significant intergroup differences in treated grape phyllosphere fungi. Correspondingly, the bacterial correlation analysis indicated a comparable pattern, with Stress values under 0.2, elucidating disparities among the bacteria on grape phyllosphere. An R value of 0.0848 and a *p*-value below 0.05 signified significant divergences among the bacteria following treatment (**Figure 2C**). To further discern the effects of varied treatments on the beta diversity of Leotiomycetes, Principal Coordinates Analysis (PCoA) was employed. The outcomes (**Figure 2D**) showed an R value of 0.2082 and a *p*-value less than 0.05, indicating the impact of the treatments on the beta diversity of the Leotiomycetes fungi.

#### 3.2.2 Correlation between fungal communities and powdery mildew indices

The redundancy analysis (RDA) revealed a clear correlation between the grape phyllosphere fungal community structure and powdery mildew disease indices in vineyards. The first two axes accounted for 27.17% and 22.02% of the variation in RDA 1 and RDA 2, respectively. This indicates the degree to which powdery mildew disease indices can influence grapevine phyllosphere fungal community structures (**Supplementary Table 1**). Over different periods, the 7-day index (*R²* = 0.790, *p* = 0.015) exerted the most significant impact on the grape’s interleaf fungal community, followed by the 14-day (*R²* = 0.785, *p* = 0.017) and 2-day indices (*R²* = 0.732, *p* = 0.023). In contrast, the initial day (0-day) index had a minimal effect (*R²* = 0.361, *p* = 0.251), From the perspective of the dominant genus, *Erysiphe* was positively correlated with the disease index at 0 days and 14 days, but not correlated with the disease index at 2 days and 7 days. *Vishniacozyma* was negatively correlated with the index at all periods, and was negatively correlated with *Erysiphe*. In general, intervals of 2, 7 and 14 days post-application significantly influenced the diversity and dynamics of grapevine phyllosphere fungal communities (*p*<0.05) (**Figure 2E**).

#### 3.2.3 Effect of *B. subtilis* on microbial community structure and composition

**Figure 3:**
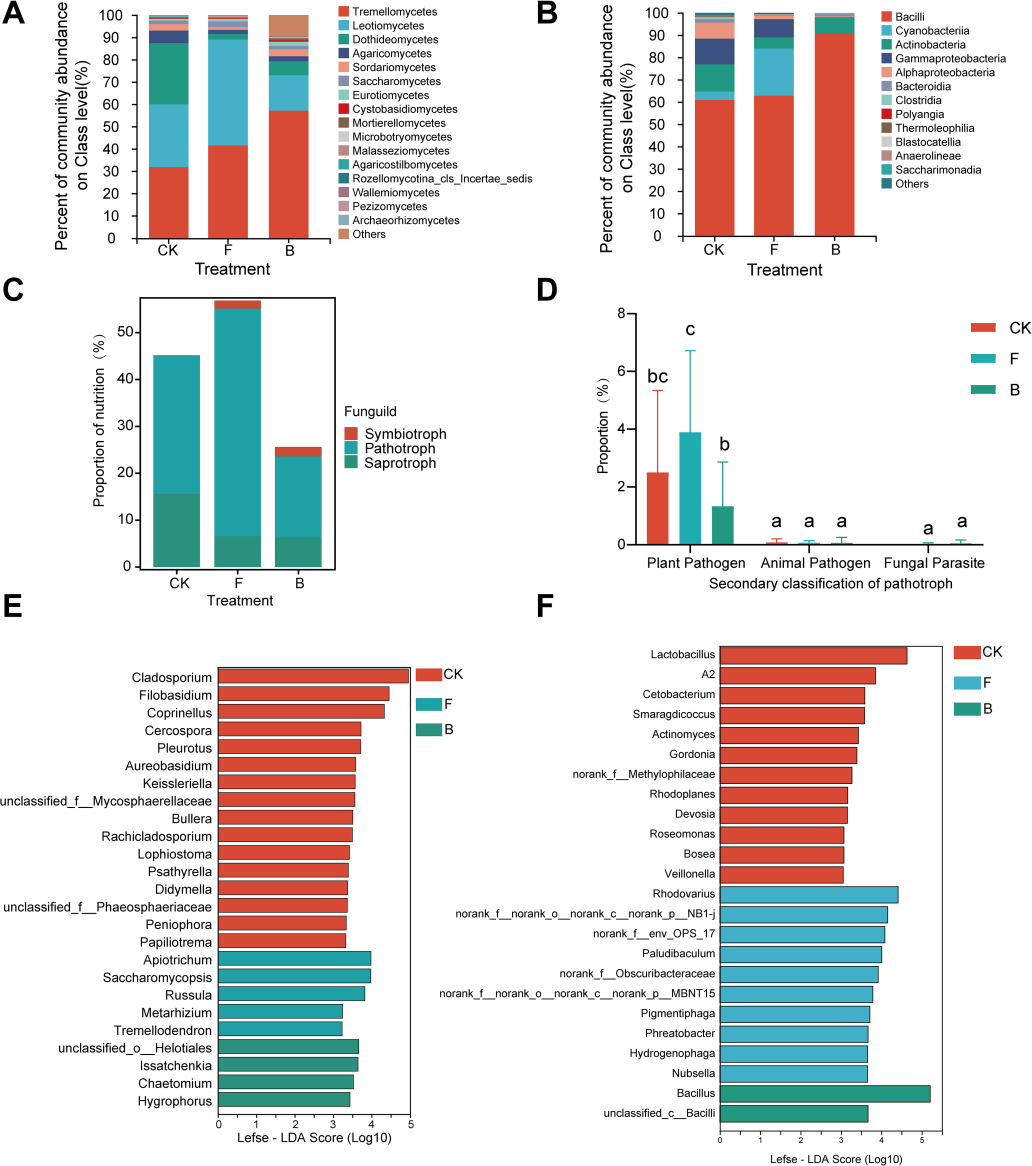
Grape phyllosphere microbial composition and fungal predictive functions under different treatments. **(A)** Analysis of dominant species composition at the fungal class level; **(B)** Analysis of dominant species composition at the bacterial class level; **(C)** Funguild functional prediction results; **(D)** Proportion of plant pathogenic fungi; **(E)** Lefse analysis results of different groups of fungal genera; **(F)** Lefse analysis results of different groups of bacterial genera.

After species annotation, a total of 455 fungal operational taxonomic units (OTUs) derived from representative sequences were classified into 6 phyla, 23 classes, 62 orders, 145 families, 240 genera, and 331 species. The analysis concentrated on the primary composition of fungal species, excluding classes with an average relative abundance below 0.1% to investigate differences at the class level. The classification demonstrated that Tremellomycetes, Leotiomycetes, and Dothideomycetes were the predominant fungal classes (**Figure 3A**), with average relative abundances of 43.48%, 30.67%, and 11.98%, respectively, together constituting 86.13% of all observations. Treatment B resulted in a 25.22% increase in the mean relative abundance of Tremellomycetes, while the mean relative abundance of Leotiomycetes decreased by 11.87% (**Supplementary Figure 2A**).

In addition, species annotation of the representative sequences identified 1,213 bacterial OTUs classified into 33 phyla, 77 classes, 187 orders, 314 families, 617 genera, and 903 species. The primary bacterial compositions were similarly assessed for species variances by excluding classes with an average relative abundance below 0.1%. At the class level, the primary bacterial constituents of the grapevine phyllosphere included Bacilli, Cyanobacteria, Actinobacteria, Gammaproteobacteria, and Alphaproteobacteria (**Figure 3B**), with mean relative abundances of 70.40%, 9.26%, 8.27%, 6.42%, and 3.14%, respectively, accounting for 97.48% of the total reads. The analysis revealed reductions in Actinobacteria, Gammaproteobacteria, and Alphaproteobacteria across both treatments, with a statistically significant decline in Alphaproteobacteria (*p* < 0.05). Conversely, Bacilli exhibited a significant increase (*p* < 0.05) in treatment B (**Supplementary Figure 2B**).

The common microbial on genera level in the three treatment groups were counted and their average relative abundance was calculated. The results showed that *Erysiphe* was predominant with average relative abundance of 40.33%, followed by *Vishniacozyma* (31.06%), *Cladosporium* (5.98%), and *Aspergillus* (1.34%). Following treatment applications, *Erysiphe* exhibited varied responses, showing minimal variance between the F and CK treatments and a decrease in the B treatment. *Vishniacozyma* and *Aspergillus* saw increases across the treatments, while *Cladosporium* declined in each instance (**Supplementary Figure 3A**). Lefse multilevel species difference discriminant analysis was used to identify genera with significant disparities at the genus level. Additionally, Lefse discriminant analysis (threshold > 4) revealed 25 key genera driving disparities in the phyllosphere fungal community, notably *Issatchenkia*, *Chaetomium*, *Hygrophorus*, and *unclassified_o_Helotiales*, which were markedly enriched in the B treatment (**Figure 3E**).

The bacterial analysis utilized a similar methodology to that of fungi, resulting in the exclusion of genera representing less than 1% relative abundance and non-bacterial entities such as chloroplasts from the preliminary analysis due to their low abundance. At the genus level, *Bacillus* emerged as the most prevalent, constituting 50.48% of relative abundance and experiencing an upsurge in the B treatment, followed by *Psychrobacillus* (8.74%), *Paenibacillus* (6.00%), and *Lactobacillus* (3.21%) (**Supplementary Figure 4**). The relative abundances of bacterial genera decreased following F and B treatments compared to the control, including the aggregate “Others” category, which encompasses numerous low-abundance taxa crucial for maintaining phyllosphere microecological stability (**Supplementary Figure 3B**). Subsequently, Lefse discriminant analysis was used to conduct genus-level analysis for all samples, identifying 24 bacterial genera with significant alterations (**Figure 3F**), among which *Bacillus* and *unclassified_c_Bacilli* were notably enriched in the B treatment (*p* <0.05).

#### 3.2.4 Prediction of nutritional patterns on fungal communities

In the study of grape leaf phyllosphere fungal communities, a total of 456 fungal (OTUs) were identified. Among these OTUs, 304, representing 66.7% of the total, were assigned to various trophic modes by Funguild. The assignment categorized 21 OTUs (4.61%) as symbiotrophic, 35 (7.68%) as pathotrophic, and 147 (32.24%) as saprotrophic. Notably, out of the 35 pathotrophic OTUs, 26 were identified as phytopathogens, constituting 74.30% of the pathogenic trophic OTUs.

The taxonomic analysis revealed distinct trophic patterns among the fungi in grape interleaf tissues across various treatments. In the control (CK) group, symbiotrophic fungi represented only 0.06%, a proportion lower than those observed in treatments F (1.72%) and B (2.14%). In contrast, saprotrophic fungi were most prevalent in the CK group, with a relative abundance of 15.57%, which was higher than the abundances recorded in treatments F (6.52%) and B (6.31%). Pathotrophic fungi exhibited the highest abundance in the F treatment group, at 48.61%, significantly surpassing the abundance in the CK group (29.55%). The B treatment exhibited the lowest abundance of pathotrophic fungi, at 17.27% (**Figure 3C**). The functional classification of pathotrophic fungi revealed a predominance of the Plant Pathogen group, particularly under the F treatment, where they represented 46.78% of the pathotrophic fungal abundance, a significantly higher percentage compared to other treatments (*p* < 0.05), followed by CK at 30.05%. The Animal Pathogen group showed the highest relative abundance in the CK group, with no significant difference noted among treatments. The abundance of the Fungal Parasite group was similar across all treatments (**Figure 3D**).

#### 3.2.5 Simper analysis of key microorganisms on the grape phyllosphere

The SIMPER analysis demonstrated that the control group (CK) had higher average relative abundances of pathogenic microorganisms such as *Erysiphe* and *Alternaria* compared to the other treatment groups. Specifically, *Erysiphe* was least abundant in treatment B, followed by treatment F. The contribution of *Erysiphe* to the observed differences between the groups was 21.58%. Conversely, *Alternaria* was more abundant in the control group, contributing 0% and 0.55% to the discrepancies between the groups. Analysis of the biocontrol bacterium *Bacillus* indicated that treatment F had the lowest relative abundance, followed by the control, while treatment B had the highest. *Bacillus* accounted for 28.75% and 36.15% of the variation between the treatment groups (**Table 2**).

**Table 2:** Analysis results of key microorganisms SIMPER under different treatments.

Genus	CK-F				CK-B			
	CK	F	SD	C R(%)	CK	B	SD	C R(%)
*Erysiphe*	44834.23	19180.67	0.14	30.28	44834.23	6121.58	0.14	21.58
*Bacillus*	56796.85	14452.25	0.15	28.75	56796.85	21233.33	0.15	36.15
*Vishniacozyma*	49794.92	15115.83	0.13	26.37	49794.92	20690.00	0.12	28.42
*Paenibacillus*	7227.31	2163.25	0.04	0.00	7227.31	3504.00	0.08	9.31
*Epicoccum*	120.62	5.25	0.00	0.10	120.62	18.75	0.00	0.11
*Solibacillus*	1750.08	626.17	0.03	1.65	1750.08	1211.25	0.04	3.22
*Staphylococcus*	312.77	12.33	0.01	0.39	312.77	1.83	0.01	0.42
*Acinetobacter*	190.23	68.75	0.00	0.28	190.23	1.17	0.00	0.18
*Lysinibacillus*	522.00	0.75	0.01	0.65	522.00	6.58	0.01	0.73

This analysis calculates the average differences between all pairs of samples and assesses the relative contribution of each species to these differences. “S D” denotes the standard deviation across different treatments; “C R” indicates the contribution rate.

### 3.3 Relationship between bacteria and fungi under different treatments

**Figure 4:**
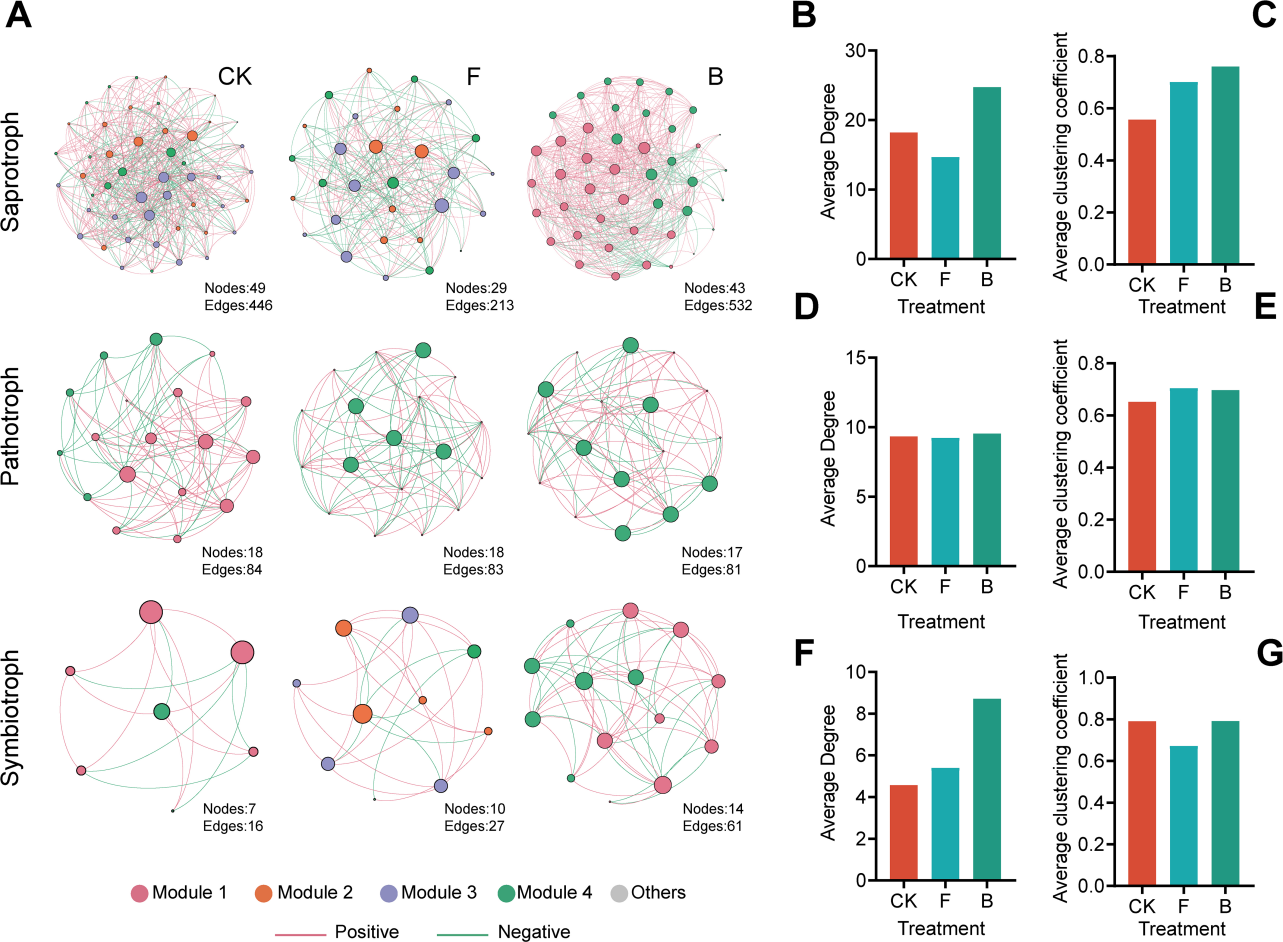
Results of the interaction network between fungi of different nutritional types and *Bacillus* bacteria. **(A)** Collinearity result diagram of the network of fungi and *Bacillus* bacteria in different trophic types; **(B, D, F)** The average degrees of the fungal networks of saprotrophic, pathotrophic, and symbiotrophic types, respectively, **(C, E, G)** The average clustering coefficients of saprotrophic, pathotrophic, and symbiotrophic types.

The fungal network analysis, as illustrated in **Figure 4A**, revealed that saprotrophic microorganisms had the highest number of correlations, followed by pathotrophic microorganisms, whereas commensal trophic microorganisms exhibited the fewest correlations. Notably, the interactions between Bacilli and the majority of saprotrophic fungi were particularly intricate, especially in the B treatment where positive correlations predominated. This was followed by the CK treatment, which displayed a mix of positive and negative correlations. Subsequent to the B treatment, the network complexity among symbiotrophic fungi experienced an increase.

The network average degree indicates that, in the analysis of fungal trophic type network interaction, the intensity of interaction is highest among saprotrophic fungi, followed by the pathogenic trophic type, with the symbiotic trophic type showing the lowest intensity (**Supplementary Table 2**). Upon analyzing the experimental groups, it was found that the average intensity after treatment B was the highest (**Figures 4B, D, F**). Furthermore, the average clustering coefficient after treatment B was slightly higher in the saprotrophic and symbiotic trophic fungal networks, but slightly lower in the pathogenic trophic fungi (**Figures 4C, E, G**).

### 3.4 Phyllosphere microbial network properties

**Figure 5:**
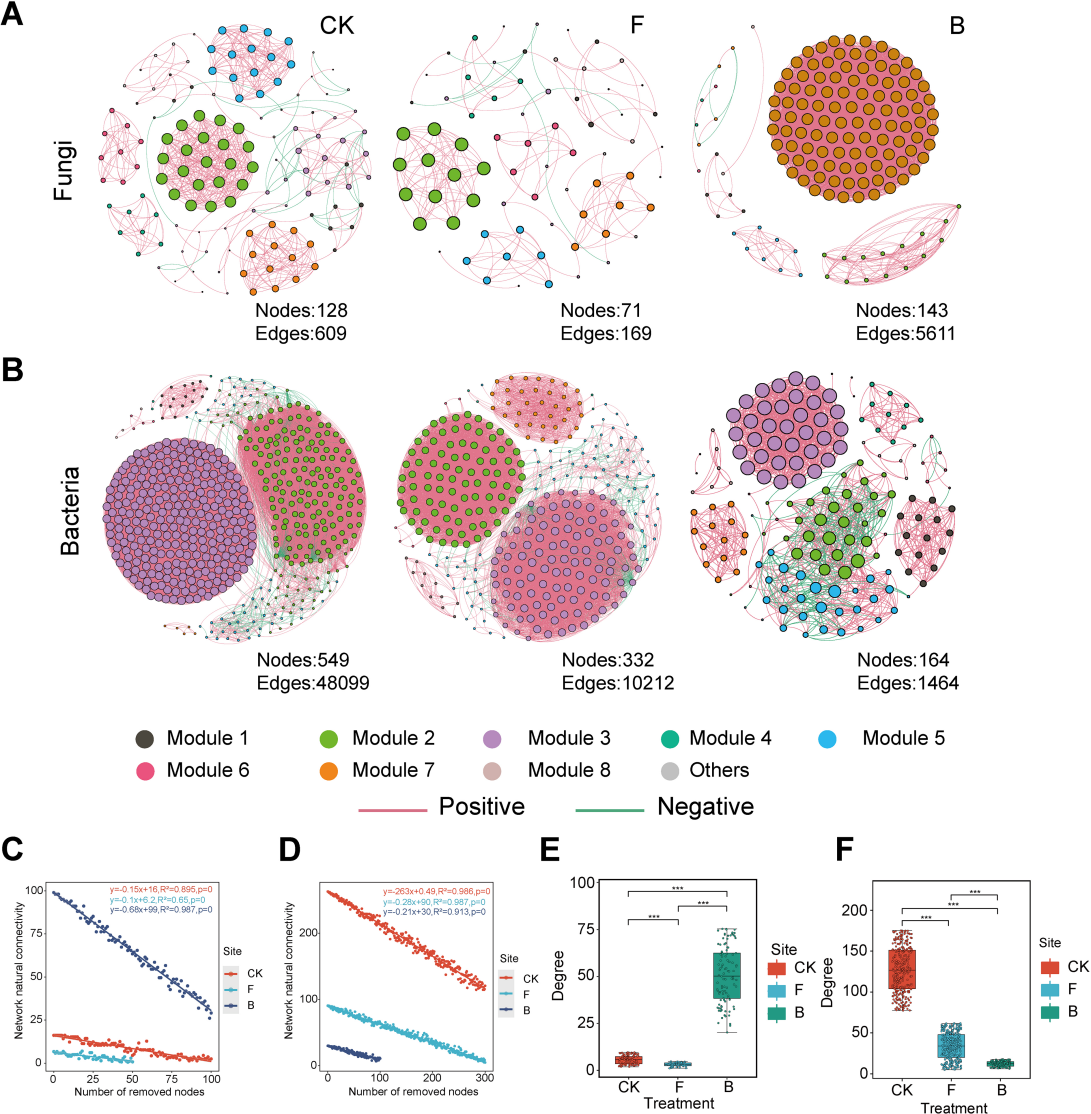
Cooccurrence Networks and Network Properties. Each node represents an individual microorganism, with “**(A)**” denoting fungi and “**(B)**” denoting bacteria. The size of the node positively correlates with its degree, and its color reflects its module. Edges depict significant Spearman correlations with p-values ranging from 0.05 to 0.7. Red lines represent positive correlations, while green lines indicate negative correlations. The network stability of the fungal **(C)** and bacterial **(D)** communities is assessed. Various colors denote different formulation treatments, and equations demonstrate changes in natural connectivity. Additionally, box plots illustrate the complexity of the fungal **(E)** and bacterial **(F)** communities, with “***” p<0.001.

A collinearity network was constructed using Spearman correlation analysis to illustrate the effects of various formulations on the structure of microbial communities in the grape phyllosphere (**Figures 5A, B**). The results revealed substantial differences in the symbiotic patterns of leaf phyllosphere microbial communities across the different formulation treatments. The topological properties presented in this study (**Figures 5E, F**) indicated a significant reduction in the complexity of fungal networks under the F treatment, while a notable increase was observed under the B treatment. In contrast, the complexity of bacterial networks significantly decreased under both the F and B treatments (*p* <0.001). Specifically, the B treatment demonstrated higher average degree, number of nodes, and number of edges in the fungal network compared to the CK and F treatments, suggesting an increase in fungal network complexity following the B treatment. However, the modularity of the fungal network was lower in the B treatment than in the F and CK treatments, indicating a potentially adverse effect on stable collinearity patterns. The F treatment exhibited the lowest average degree, fewest nodes and connections, and highest modularity in the fungal network. Regarding the bacterial network, the topological indices revealed that the B treatment led to reductions in average degree, number of nodes, and number of edges, while achieving the highest modularity. Conversely, the CK treatment recorded the highest average degree, number of nodes, and connections, but the lowest modularity in the bacterial network (**Supplementary Table 3**). Furthermore, network stability was evaluated by assessing the impact of node removal on connectivity. For the fungal network, the B treatment resulted in the most significant decrease in natural connectivity, indicating the lowest stability (**Figure 5C**). In the bacterial network, the CK treatment exhibited the most substantial decrease in natural connectivity, while the B treatment showed the highest natural connectivity, indicating the greatest stability (**Figure 5D**).

### 3.5 The functions of key microorganisms on the grape phyllosphere

**Figure 6:**
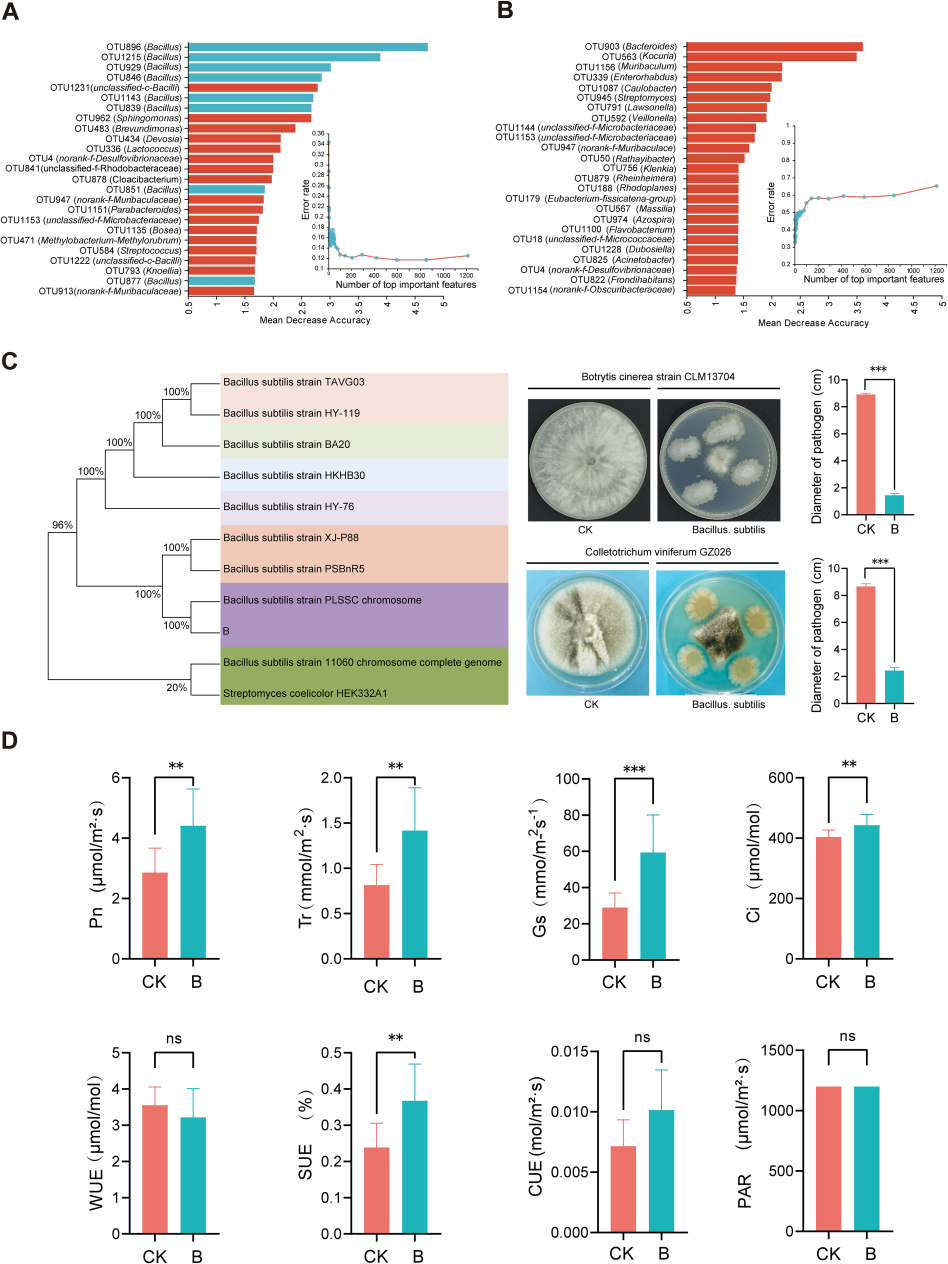
Antibacterial effect of main microorganisms (*B. subtilis*) on grape phyllosphere and its effect on photosynthesis of grape leaves. The results of key OTUs and annotated species screened by Random forest (RF) were validated using cross-validation (**A**, *B. subtilis;* **B**, Mixed fungicide). After the *B*. *subtilis* treatment, the capability of isolated and cultured microorganisms on the phyllosphere to antagonize grape pathogens was assessed **(C)**. **(D)**: Effects of *B*. *subtilis* treatment on photosynthetic parameters of grape leaves. The symbol “ns” indicates no significant difference after using the Student’s T-test (T-test) method (*p*>0.05), “**” denotes *p*<0.01, and “***” indicates *p*<0.001.

A random forest analysis was conducted on all OTUs in the B/F treatment, ranking the top 25 important results (**Supplementary Table 4** and **Table 5**). The findings indicated that following the B treatment, the top 4 OTUs were OTU896, OTU1215, OTU929, and OTU846, all annotated as *Bacillus* (**Figure 6A**). Among the 25 important OTU rankings, 8 were annotated as *Bacillus*. The model was validated through cross-validation. In the F treatment, the top-ranked OTU was OTU903, identified as *Bacteroides*, with no repeated OTUs in the top 25 (**Figure 6B**). The main bacteria isolated in treatment B were identified as *B. subtilis*, which was found to significantly inhibit *Colletotrichum viniferum* GZ026 and *Botrytis cinerea* strain CLM13704 (*p* < 0.001) (**Figure 6C**).

Grape seedlings were cultured in the growth chamber and inoculated with *B. subtilis*, following which changes in photosynthetic indicators were measured. The results demonstrated that after *Bacillus subtilis* treatment, the net photosynthetic rate (Pn), transpiration rate (Tr), stomatal conductance (Gs), intercellular CO_2_ concentration (Ci), and instantaneous light energy use efficiency (SUE) of grape leaves were significantly higher than those of the control group (*p* < 0.01). However, no significant changes were observed in the indicators of instantaneous water use efficiency (WUE), photosynthetically active radiation (PAR), and instantaneous carboxylation efficiency (CUE) (**Figure 6D**).

## 4 Discussions

Our research provides the first comprehensive analysis of the primary impacts of biocontrol agents and fungicides on the microbial community structure of grapevine phyllospheres and their efficacy in managing grape powdery mildew. This innovative assessment of their combined influence on fungal communities and grape leaf pathogens offers novel insights. Field trials showed that both biocontrol agent treatment B and fungicide treatment F could effectively inhibit grape powdery mildew. At the same time, the analysis of microbial community structure and composition found that the relative abundance of beneficial bacteria increased and powdery mildew decreased after biocontrol agent treatment, which may be one of the reasons affecting the prevalence of the disease.

### 4.1 Grape phyllosphere microbial diversity changes with biocontrol bacteria

Plant leaves serve as the primary location for photosynthesis and energy metabolism, contributing significantly to the growth and developmental processes of plants (Zhu et al., 2023). The plant phyllosphere is inhabited by a diverse array of microorganisms, and higher microbial diversity has been linked to improved coordination within the leaf microbial community structure, resulting in greater stability in the leaf microbiota (Chen et al., 2020). The use of chemical agents to control grape powdery mildew can also impact the structure and diversity of the microbial community on grape leaves, leading to a reduction in harmful bacterial populations. Our study found that fungicide treatment significantly reduced bacterial community *α* diversity but had no significant impact on the fungal community (*p* <0.05), this is similar to the results of previous studies showing a decrease in microbial diversity on citrus leaves after the application of biocontrol bacteria (Li et al., 2020a). The results also showed that biological control agents did not reduce the diversity of grape phyllosphere bacterial and fungal communities, which is similar to the previous results of the effects of chemical and biological fungicides on grape biofilm microorganisms (Escribano-Viana et al., 2018).

### 4.2 Biocontrol bacteria increase grape phyllosphere microbial beneficial bacterial community

The application of various bacterial agents can impact the homeostasis of this microbiota. Typical bacterial and fungal communities inhabit grape leaves, particularly the abundant fungal communities, including Tremellomycetes, Leotiomycetes, and Dothedeomycetes. The prevalent bacteria classes primarily consist of Bacilli, Cyanobacteria, and Actinobacteria. Treatment B enriched the *Bacillus*. The *Bacillus*, classified within Bacilli, is known as a prospective biocontrol agent, effectively outcompeting some harmful bacteria (Miljaković et al., 2020). *Bacillus* can enhance the net photosynthetic rate and stomatal conductance of plant leaves and can suppress phytopathogenic bacteria by facilitating nutrient uptake and providing growth and development-enhancing compounds to plants (Borriss, 2011). They produce antibiotics (Ongena and Jacques, 2008) and cell wall hydrolases (Maachia et al., 2015). *Bacillus* spp. also inhibit the growth of competing microorganisms in the plant rhizosphere and promote plant growth by facilitating iron uptake and enhancing biocontrol activity (Goswami et al., 2016). Sterile inoculation of *Bacillus.* spp. strains significantly increases the fresh and dry biomass, plant height, photosynthetic pigments, and biochemical traits in maize cultivars (*p* <0.05) (Azeem et al., 2022). *B. megaterium* and *B. saffron* boost plant growth parameters, including root and stem dry weights and seed weight of wheat during field trials (Mukhtar et al., 2017). *B. subtilis*, a predominant bacterial genus in the soil, is able to produce Indoleacetic acid (IAA), positively influencing onion growth and yield (Čolo et al., 2014). With its vast genetic and metabolic diversity, *Bacillus* thrives in diverse environmental conditions, making it a suitable candidate for biocontrol agents. In this study, treatments with *Bacillus*-based biocontrol agents effectively controlled the field disease index of grapevine powdery mildew. Microbial sequencing confirmed that these treatments indeed increased the relative abundance of *Bacillus*. Network analysis further revealed that *Bacillus*-like bacteria predominantly exhibited positive correlations with saprophytic fungi, showing extensive associations with commensal microorganisms across the three treatments.

In the B treatment, significant enrichment of *Issatchenkia*, *Chaetomium*, and *Hygrophorus* was observed. *Issatchenkia*, a genus of saccharomycetes, is notable for its resilience in low pH environments and its ability to produce citrate, as demonstrated by *Issatchenkia orientalis* (Wu et al., 2023). Further research highlights that *Issatchenkia orientalis* having a high biotechnological value and being able to act as an accelerator to speed up the composting process (Da Silva Gaspar et al., 2023). *Chaetomium* plays a defensive role against plant pathogens; for example, *Chaetomium cochliodes* exhibits substantial antagonistic effects on the pathogen *Marssonina rosae*, achieving an inhibition rate of 60.7% and a relative inhibition rate of 5.38 after a 72-hour incubation (*p* <0.01). This indicates its potential as a biocontrol agent against rose black spot disease (Pang et al., 2023). Additionally, *Chaetomium globosum*, a prevalent plant species, has been shown to delay the appearance of stem base browning symptoms in wheat blast (*Fusarium* crown rot, FCR) and substantially lower the disease index (Feng et al., 2023).

The B treatment increased the number of beneficial microbial species is more than the F treatment. However, the F treatment Enrich the fungi *Apiotrichum*, *Saccharomycopsis*, *Russula*, *Metarhizium*, and *Tremellodendron*. Among these, *Apiotrichum*, *Saccharomycopsis* are saccharomycetes, and *Metarhizium* has been reported as a pathogenic fungus capable of triggering the death of harmful insects (Dubois et al., 2023). There are fewer studies on *Tremellodendron*.

### 4.3 Biocontrol bacteria affect microbial collinearity networks

Microorganisms play a vital role in the microecosystem of plant leaves, engaging in a variety of interactions with other species. These interactions can occur at different times and contribute to the establishment of a three-dimensional interactive microbial network (Karimi et al., 2017). Furthermore, microbial networks offer a unique perspective on ecosystems (Huang et al., 2023). The principle underlying co-occurrence networks involves connecting significantly correlated species by calculating correlation coefficients between them. In comparison to the control group, the number of edges in the fungal symbiotic network increased following treatment B, suggesting that this treatment facilitated the establishment of cooperative connections among fungal species. In contrast, the bacterial network exhibited an opposing trend after treatment B, with the number of edges in the bacterial symbiotic network being lower than that in the control group. Modules typically represent functional units within various ecological processes (Zhang et al., 2018). An increase in the number of modularized microbial networks indicates that exposure to specific environmental factors has led the microbial community to develop more complex ecological niches and functional units (Wang et al., 2021). Studies have shown that the functions and trophic strategies of similar species tend to cluster within the same module, which features a greater number of internal connections than external ones, thereby limiting external interference (Lurgi et al., 2019; Hernandez et al., 2021). Compared to the control group, the fungal symbiotic network post-treatment B exhibited lower modularity, suggesting a suppression of microbial diversity and reduced network stability. Previous research has shown that network modularity is influenced by complexity, where increased connectivity can boost core connections and reduce modularity levels (Olesen et al., 2007). This might be attributed to the colonization of numerous single *Bacillus* species occupying specific ecological niches post-treatment B. Conversely, the modularity degree of the bacterial symbiotic network post-treatment B was higher than the control group, indicating greater network density. These findings suggest that treatment B promoted cooperative connections among fungal species while enhancing modularity among bacteria, creating a more detailed and beneficial “*Bacillus* microenvironment” ecological niche. On the grape phyllosphere microbial symbiosis network, the clustering coefficient signifies node connections, with a higher value indicating greater importance of the node. In this investigation, treatment B raised the average clustering coefficient of symbiotic bacteria, indicating that B treatment encourages interconnections among bacterial communities. Furthermore, results from the robustness test based on the natural connectivity of removed nodes revealed that treatment B positively influenced the bacterial network’s stability.

### 4.4 Functional adaption of the microbial community

Plant beneficial microorganisms can impact plant growth through direct or indirect mechanisms (Elnahal et al., 2022). They can influence plant nutrition, energy absorption, induce plant disease resistance, synthesize growth hormones, and more to foster plant growth. Additionally, some beneficial bacteria have the capability to produce antibiotics that directly target pathogen cell walls, thus enhancing disease resistance (Nazari and Smith, 2020). In this study, a significant accumulation of *Bacillus* was observed on grape leaves following treatment B, which resulted in a notable reduction in the disease index of grape field diseases. This microenvironment plays a crucial role in the control of plant pathogens through both direct and indirect mechanisms.

A substantial amount of *B. subtilis* was isolated from the grape phyllosphere in our research, showing significant potential for agricultural applications and effective inhibition of plant diseases caused by fungi, bacteria, among others (Etesami et al., 2023). The study findings align with prior research, demonstrating the significant suppression of grape powdery mildew occurrence when using this type of biocontrol bacteria on foliage. Previous studies have illustrated that a combined preparation of *B. subtilis* and *B. thuringiensis* can enhance the synthesis of plant defense enzymes, indirectly managing cucurbit powdery mildew (Ünlü et al., 2023). These mechanisms may also apply to grapevine disease control. Furthermore, the application of *B. subtilis* on grape leaves was found to positively regulate photosynthetic indicators. Results indicated that *B. subtilis* effectively improves grape photosynthetic performance, leading to a significant increase in the net photosynthetic rate (Pn), transpiration rate (Tr), instantaneous light energy utilization rate (SUE), and overall photosynthetic capacity. These outcomes are consistent with recent studies on microorganisms impacting tomato growth (Liu et al., 2024).

Microorganisms play a pivotal role in shaping phyllosphere microbiota communities mediating plant-microbe interation, and modulating their own population dynamics, thus orchestrating the complex web of host-microbal relationships (Trivedi et al., 2020). Although chemical agents are employed for diseases prevent and control, they non-selective nature may adversely affect non-target organisms within the leaf ecosystem, including beneficial species. Simultaneously, inherent ecological restoration capabilities of the phyllosphere are engaged. Predictive microbial function analysis, while informative, offers limited insights, necessitating integrated approaches such as transcriptomic and metagenomic analyses for precise indentification of microbial functions influenced by different treatment. The administration of the different treatments in question invariably impacts the equilibrium of the leaf microbiota to some extent. When juxtaposed with conventional microbial study techniques, high-throughput sequencing, particularly Illumina-based platforms, provides a more detailed representation of microbial relative abundance and shifts across various taxonomic strata. In synergy with advancements in bioinformatics, engineered plant-microbe interaction system, among other innovations, this approach lays a robust foundation for enhancing plant health via microbial regulation in the context of modern agriculture.

## 5 Conclusion

In conclusion, our study demonstrates that fungicidal treatments decrease the diversity and alter the composition of bacterial communities on plant phyllosphere microbiota, while biocontrol agents have minimal comparative effects. Importantly, these agents can also reduce the relative abundance of plant pathogens, highlighting the effectiveness of biocontrol bacteria. Moreover, biocontrol agents can establish a *Bacillus* microenvironment, leading to the proliferation of beneficial microorganisms. Additionally, biocontrol bacteria improve connections with symbiotic microorganisms, enhance bacterial network modularity, create a more detailed *Bacillus* ecological niche, boost plant photosynthetic ability, thereby sustaining phyllosphere microbial community balance and fostering plant growth. Based on these results, it is advisable to implement a strategic integration of fungicides and biological control agents in vineyard management within this grape growing region, as this approach is more conducive to preserving grape quality and phyllosphere microbiota stability.

## Data Availability

The datasets presented in this study can be found in online repositories. The names of the repository/repositories and accession number(s) can be found in the article/**Supplementary Material**.
