# Supplementary material for: Biocontrol agents transform the stability and functional characteristics of the grape phyllosphere microenvironment
Source: Front Plant Sci. 2024 Oct 16;15:1439776. doi: 10.3389/fpls.2024.1439776 (PMC11524152; doi:10.3389/fpls.2024.1439776)
Supplement: Supplementary file 1 [file DataSheet1.docx]

***Supplementary Material***

**Figure S1** The graph illustrates the diluted curves of Shannon diversity indices for fungal (A) and bacterial (B) microorganisms in the phyllosphere of grape leaves under varying treatments. The x-axis denotes the sequencing data volume randomly selected, while the y-axis represents the observed species’ Shannon diversity index.

**Figure S2** The identification of species exhibiting significant variations at the order level within fungal (A) and bacterial (B) communities in the phyllosphere of grape leaves under different treatments was conducted. The statistical analysis involved employing the Kruskal-Wallis H test, with the FDR method utilized for multiple testing correction. Post-hoc tests were subsequently employed for further investigation, employing a 95% confidence interval. Statistical significance was indicated as * *p* < 0.05, ** *p* < 0.01, *** *p* < 0.001.

**Figure S3** The composition of fungal (A) and bacterial (B) communities on the phyllosphere of grape leaves under different treatments.

**Figure S4** Analysis of variations in abundance of bacilli on the phyllosphere of grape leaves under distinct treatments. The Kruskal-Wallis H test was utilized to examine the statistical significance, with multiple testing correction employing the FDR method. Post-hoc tests were subsequently conducted to obtain further insights, employing a 95% confidence interval. Significance levels were denoted as * *p* < 0.05, ** *p* < 0.01, *** *p* < 0.001.

**Table S1** Analysis results of powdery mildew pathogen, powdery mildew disease index, and microbial rda at different time periods. The original data was standardized using the Hellinger method, and RDA analysis was performed utilizing the Abundance sorting method. The statistical test employed was the Permutation Test, with a closer R2 value to 1 indicating a higher level of model fitness. A significance level of *p* < 0.05 denoted the presence of significant differences.

**Table S2** Network collinearity index for fungi of different trophic types.

**Table S3** Collinearity index of fungal and bacterial networks under different treatments.

**Table S4** Results of the random forest importance ranking for fungal (OTUs).

**Table S5** Results of the random forest importance ranking for bacteria (OTUs).


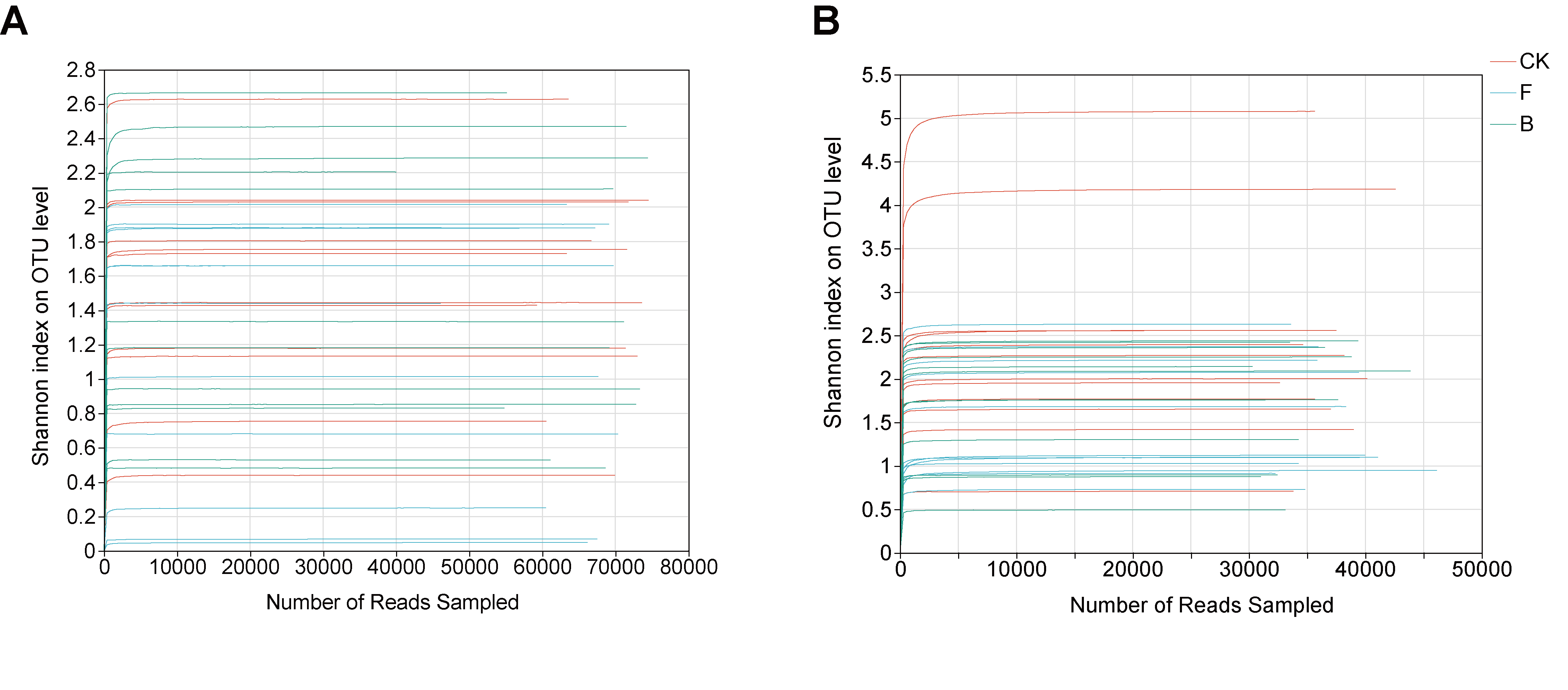


**Figure S1** The graph illustrates the diluted curves of Shannon diversity indices for fungal (A) and bacterial (B) microorganisms in the phyllosphere of grape leaves under varying treatments. The x-axis denotes the sequencing data volume randomly selected, while the y-axis represents the observed species’ Shannon diversity index.


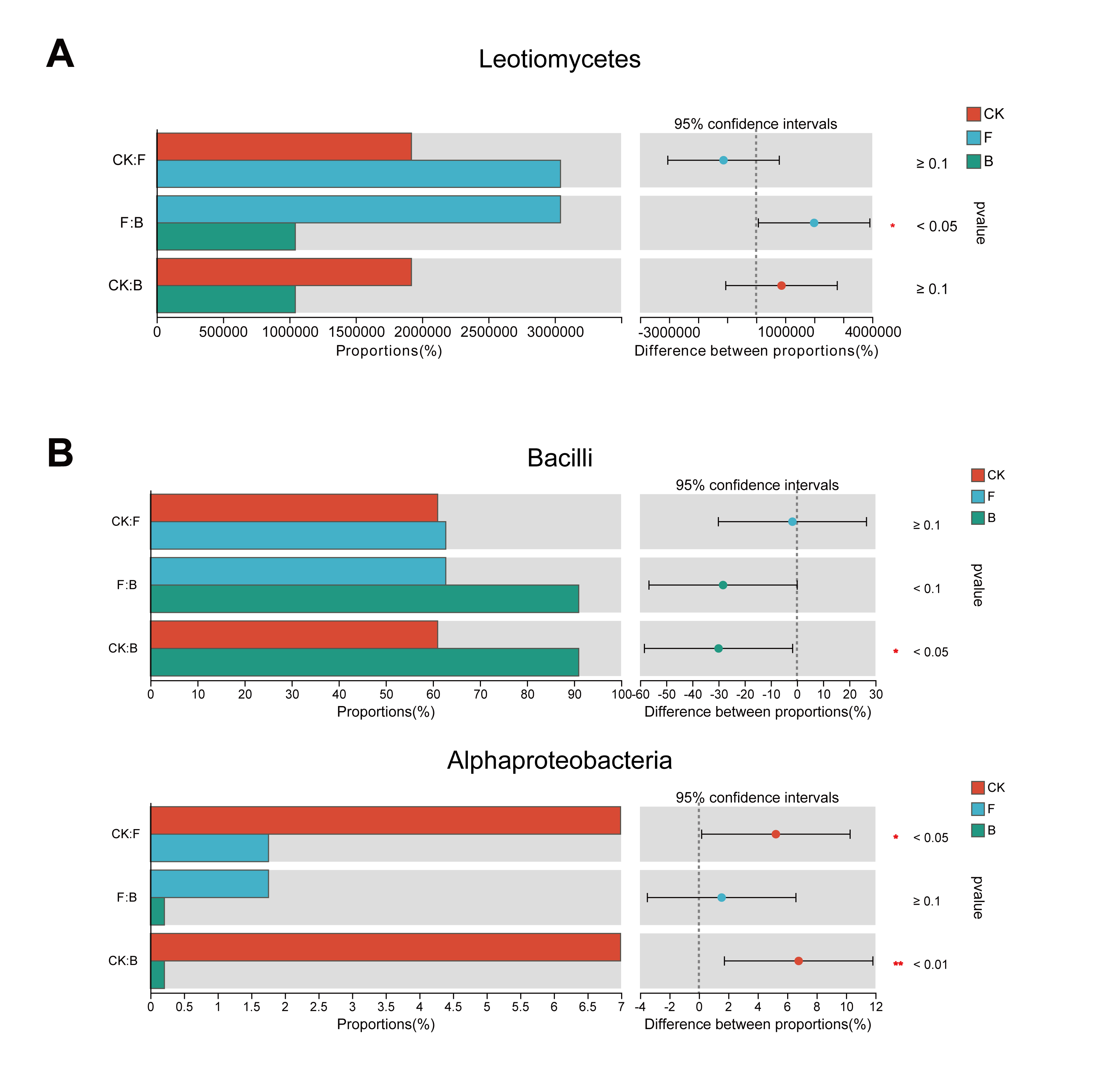


**Figure S2** The identification of species exhibiting significant variations at the order level within fungal (A) and bacterial (B) communities in the phyllosphere of grape leaves under different treatments was conducted. The statistical analysis involved employing the Kruskal-Wallis H test, with the FDR method utilized for multiple testing correction. Post-hoc tests were subsequently employed for further investigation, employing a 95% confidence interval. Statistical significance was indicated as * *p* < 0.05, ** *p* < 0.01, *** *p* < 0.001.


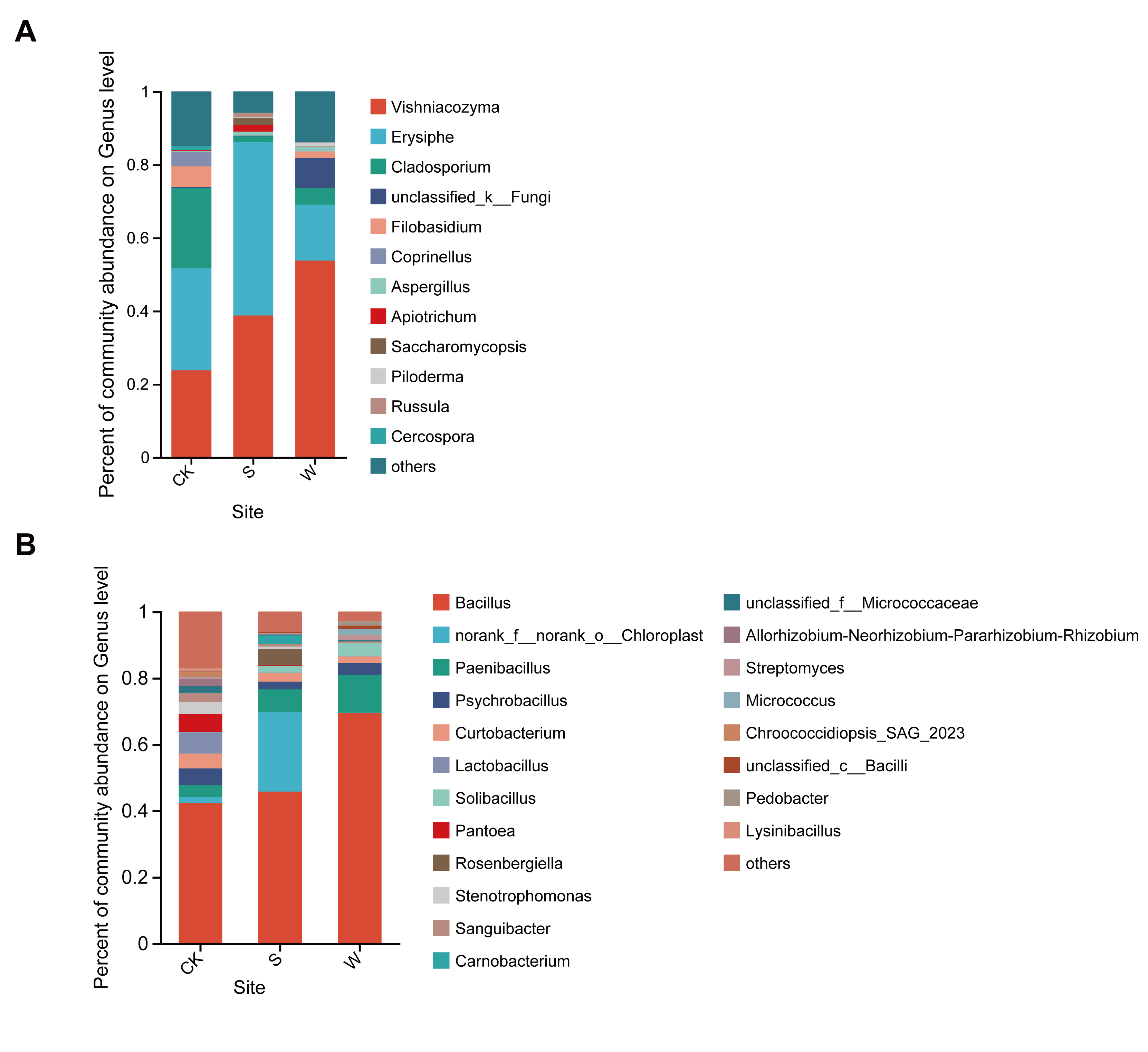


**Figure S3** The composition of fungal (A) and bacterial (B) communities on the phyllosphere of grape leaves under different treatments.


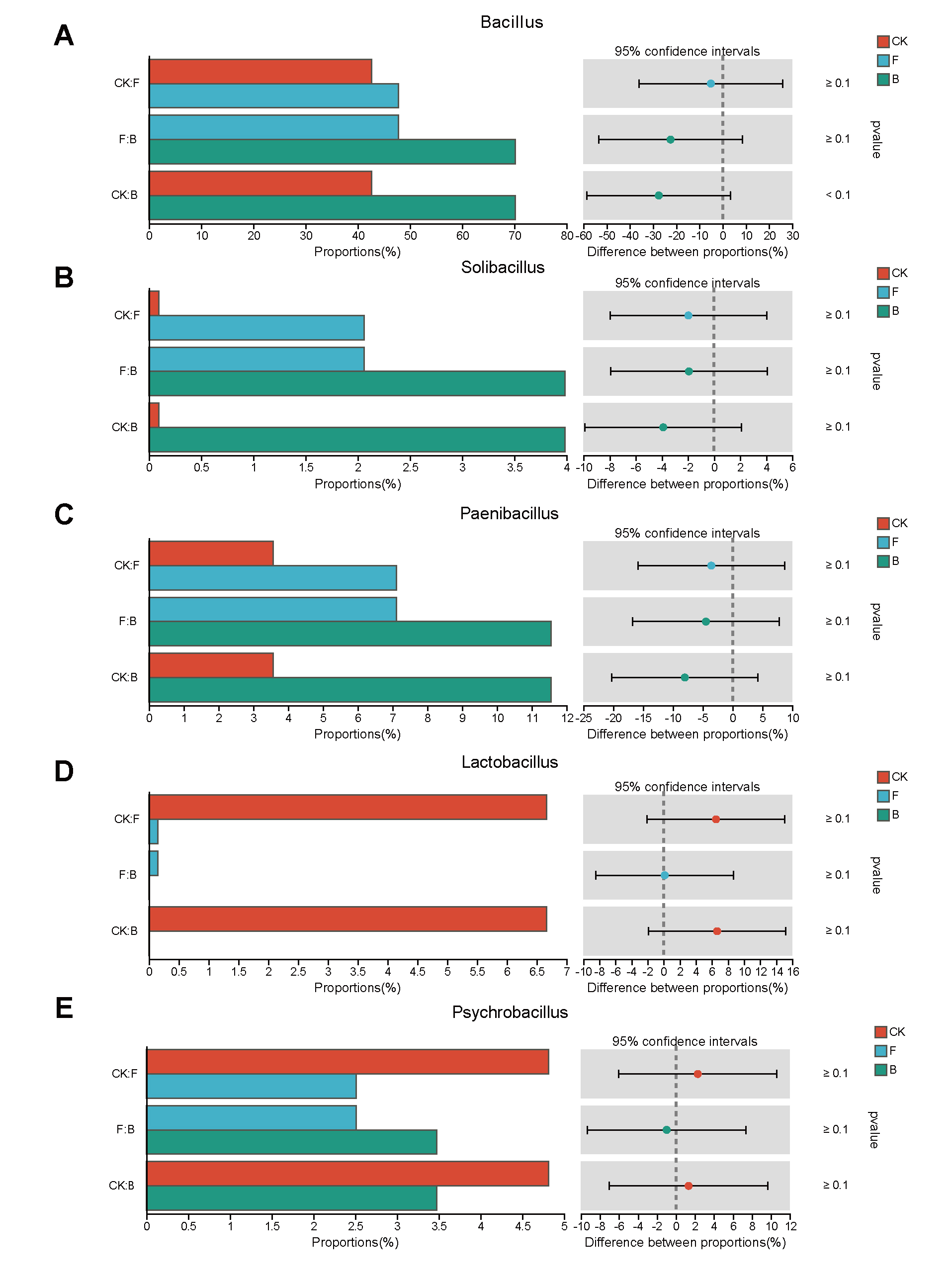


**Figure S4** Analysis of variations in abundance of bacilli on the phyllosphere of grape leaves under distinct treatments. The Kruskal-Wallis H test was utilized to examine the statistical significance, with multiple testing correction employing the FDR method. Post-hoc tests were subsequently conducted to obtain further insights, employing a 95% confidence interval. Significance levels were denoted as * *p* < 0.05, ** *p* < 0.01, *** *p* < 0.001.

**Table S1** Analysis results of powdery mildew pathogen, powdery mildew disease index, and microbial rda at different time periods. The original data was standardized using the Hellinger method, and RDA analysis was performed utilizing the Abundance sorting method. The statistical test employed was the Permutation Test, with a closer R2 value to 1 indicating a higher level of model fitness. A significance level of *p* < 0.05 denoted the presence of significant differences.

| **Time** | **RDA1** | **RDA2** | **R^2^** | **p** |
| --- | --- | --- | --- | --- |
| **0d** | 0.430252 | -0.902709 | 0.361721 | 0.28 |
| **2d** | 0.686705 | 0.726936 | 0.732117 | 0.019 |
| **7d** | 0.710821 | 0.703373 | 0.790332 | 0.014 |
| **14d** | 0.850521 | 0.525941 | 0.785469 | 0.016 |

**Table S2** Network collinearity index for fungi of different trophic types

| **Nutritional type** | **Group** | **Number of nodes** | **Number of edges** | **Average Degree** | **Average weighting** | **Number of modules** | **Modularity** | **Average clustering coefficient** |
| --- | --- | --- | --- | --- | --- | --- | --- | --- |
| **Saprotroph** | **CK** | 49 | 446 | 18.204 | 18.204 | 3 | 0.152 | 0.556 |
|  | **F** | 29 | 213 | 14.69 | 14.69 | 3 | 0.107 | 0.701 |
|  | **B** | 43 | 532 | 24.744 | 24.744 | 2 | 0.136 | 0.76 |
| **Pathotroph** | **CK** | 18 | 84 | 9.333 | 9.333 | 2 | 0.113 | 0.652 |
|  | **F** | 18 | 83 | 9.222 | 9.222 | 2 | 0.112 | 0.705 |
|  | **B** | 17 | 81 | 9.529 | 9.529 | 2 | 0.129 | 0.697 |
| **Symbiotroph** | **CK** | 7 | 16 | 4.571 | 4.571 | 2 | 0 | 0.79 |
|  | **F** | 10 | 27 | 5.4 | 5.4 | 3 | 0.097 | 0.672 |
|  | **B** | 14 | 61 | 8.714 | 8.714 | 2 | 0.114 | 0.791 |

**Table S3** Collinearity index of fungal and bacterial networks under different treatments

| **Nutritional type** | **Group** | **Number of nodes** | **Number of edges** | **Average Degree** | **Average weighting** | **Number of modules** | **Modularity** | **Average clustering coefficient** |
| --- | --- | --- | --- | --- | --- | --- | --- | --- |
| **Fungal** | **CK** | 128 | 609 | 9.516 | 9.516 | 11 | 0.774 | 0.837 |
|  | **F** | 71 | 169 | 4.761 | 4.761 | 13 | 0.794 | 0.88 |
|  | **B** | 143 | 5611 | 78.476 | 78.476 | 8 | 0.053 | 0.999 |
| **Bacterial** | **CK** | 175.224 | 175.224 | 8 | 0.373 | 0.903 | 175.224 | 175.224 |
|  | **F** | 61.518 | 61.518 | 7 | 0.568 | 0.855 | 61.518 | 61.518 |
|  | **B** | 17.854 | 17.854 | 11 | 0.667 | 0.828 | 17.854 | 17.854 |

**Table S4** Results of the random forest importance ranking for fungal (OTUs)

| **Feature** | **Importance** | **Genus** |
| --- | --- | --- |
| OTU124 | 6.096741252 | *g__unclassified_f__Mycosphaerellaceae* |
| OTU291 | 5.832944345 | *g__Vishniacozyma* |
| OTU242 | 5.74142183 | *g__Saccharomycopsis* |
| OTU265 | 5.708444959 | *g__Vishniacozyma* |
| OTU30 | 5.556792458 | *g__Apiotrichum* |
| OTU273 | 5.167473755 | *g__Erysiphe* |
| OTU15 | 4.956146461 | *g__Cercospora* |
| OTU214 | 4.845391097 | *g__Russula* |
| OTU312 | 4.748547484 | *g__Filobasidium* |
| OTU68 | 4.68266106 | *g__Aureobasidium* |
| OTU268 | 4.644993003 | *g__Vishniacozyma* |
| OTU294 | 3.956103158 | *g__Chaetomium* |
| OTU274 | 3.903987285 | *g__Schizophyllum* |
| OTU257 | 3.889563826 | *g__Cutaneotrichosporon* |
| OTU313 | 3.809801331 | *g__Cladosporium* |
| OTU298 | 3.759305913 | *g__Acremonium* |
| OTU41 | 3.421019147 | *g__Zygosaccharomyces* |
| OTU13 | 3.192969183 | *g__Symmetrospora* |
| OTU295 | 2.694316458 | *g__Saccharomyces* |
| OTU6 | 2.675262561 | *g__Didymella* |
| OTU42 | 2.390420536 | *g__Psathyrella* |
| OTU201 | 2.370646941 | *g__Vishniacozyma* |
| OTU142 | 2.333605922 | *g__Peniophora* |
| OTU24 | 2.182242636 | *g__Lophiostoma* |
| OTU145 | 2.111073149 | *g__Bullera* |
| OTU105 | 2.059149743 | *g__Papiliotrema* |
| OTU113 | 1.953818344 | *g__Rachicladosporium* |
| OTU404 | 1.73727046 | *g__Issatchenkia* |
| OTU103 | 1.735093167 | *g__Cladosporium* |
| OTU266 | 1.733501781 | *g__unclassified_o__Pleosporales* |
| OTU175 | 1.729442713 | *g__Wallemia* |
| OTU87 | 1.714696819 | *g__Zygosporium* |
| OTU511 | 1.694811263 | *g__Sporidiobolus* |
| OTU238 | 1.610933094 | *g__Malassezia* |
| OTU1 | 1.415913679 | *g__Cladosporium* |
| OTU9 | 1.392255057 | *g__Pleurotus* |
| OTU27 | 1.357063236 | *g__Erythrobasidium* |
| OTU157 | 1.357063236 | *g__Mortierella* |
| OTU40 | 1.34406228 | *g__unclassified_f__Cystobasidiaceae* |
| OTU476 | 1.334274573 | *g__Epicoccum* |
| OTU269 | 1.317443759 | *g__Alternaria* |
| OTU247 | 1.001001503 | *g__Talaromyces* |
| OTU230 | 1.001001503 | *g__unclassified_p__Ascomycota* |
| OTU292 | 1.001001503 | *g__Mortierella* |
| OTU63 | 1.001001503 | *g__Penicillium* |
| OTU112 | 1.001001503 | *g__unclassified_f__Phaeosphaeriaceae* |
| OTU93 | 1.001001503 | *g__unclassified_f__Trichomeriaceae* |
| OTU335 | 1.001001503 | *g__Trechispora* |
| OTU158 | 1.001001503 | *g__Candida* |
| OTU19 | 1.001001503 | *g__Keissleriella* |
| OTU34 | 1.001001503 | *g__unclassified_o__Hypocreales* |
| OTU177 | 1.001001503 | *g__Aspergillus* |
| OTU490 | 1.001001503 | *g__Cystobasidium* |
| OTU106 | 1.001001503 | *g__unclassified_f__Peniophoraceae* |
| OTU89 | 1.001001503 | *g__unclassified_f__Phaeosphaeriaceae* |
| OTU130 | 1.001001503 | *g__Trametes* |
| OTU391 | 1.001001503 | *g__Candida* |
| OTU495 | 1.001001503 | *g__Cryptococcus_f__Tremellaceae* |
| OTU310 | 1.001001503 | *g__Diutina* |
| OTU75 | 1.001001503 | *g__Tetraplosphaeria* |
| OTU62 | 1.001001503 | *g__Neocosmospora* |
| OTU223 | 0.968468978 | *g__Metarhizium* |
| OTU241 | 0.890166085 | *g__Tremellodendron* |
| OTU317 | 0.878558401 | *g__Piloderma* |
| OTU235 | 0.832964788 | *g__Aspergillus* |
| OTU248 | 0.748790876 | *g__Botrytis* |
| OTU188 | 0.62451665 | *g__Malassezia* |
| OTU10 | 0.61921485 | *g__Coprinellus* |
| OTU504 | 0.346546826 | *g__Aspergillus* |
| OTU311 | 0.241654259 | *g__Rhodotorula* |
| OTU271 | 0.136225511 | *g__Symmetrospora* |
| OTU478 | 0.117445664 | *g__Fusarium* |
| OTU20 | 0.100759567 | *g__Paraphaeosphaeria* |
| OTU143 | 0.056523522 | *g__Paraphoma* |
| OTU8 | 0.056523522 | *g__unclassified_f__Didymellaceae* |
| OTU165 | 0.052342536 | *g__Filobasidium* |
| OTU329 | 0.042835372 | *g__Cutaneotrichosporon* |
| OTU288 | 0.029501246 | *g__Nigrospora* |

**Table S5** Results of the random forest importance ranking for bacteria (OTUs)

| **Feature** | **Importance** | ***Genus*** |
| --- | --- | --- |
| OTU1215 | 4.347514295 | *g__Bacillus* |
| OTU903 | 4.033736194 | *g__Bacteroides* |
| OTU896 | 3.893701266 | *g__Bacillus* |
| OTU1014 | 3.886345301 | *g__norank_f__norank_o__Chloroplast* |
| OTU563 | 3.861479649 | *g__Kocuria* |
| OTU929 | 3.627041039 | *g__Bacillus* |
| OTU839 | 3.352520225 | *g__Bacillus* |
| OTU864 | 3.301613846 | *g__Bacillus* |
| OTU1143 | 3.049389973 | *g__Bacillus* |
| OTU846 | 2.877923971 | *g__Bacillus* |
| OTU1191 | 2.790307642 | *g__Bacillus* |
| OTU592 | 2.718984633 | *g__Veillonella* |
| OTU1153 | 2.59221089 | *g__unclassified_f__Microbacteriaceae* |
| OTU434 | 2.585507988 | *g__Devosia* |
| OTU962 | 2.298039541 | *g__Sphingomonas* |
| OTU3 | 2.253312151 | *g__Faecalibaculum* |
| OTU1158 | 2.22451358 | *g__Paenibacillus* |
| OTU1171 | 2.212359079 | *g__Micrococcus* |
| OTU1163 | 2.192056588 | *g__Paenibacillus* |
| OTU963 | 2.185733143 | *g__Brachybacterium* |
| OTU19 | 1.978279958 | *g__Paenarthrobacter* |
| OTU851 | 1.838881383 | *g__Bacillus* |
| OTU783 | 1.796792257 | *g__Massilia* |
| OTU877 | 1.775596942 | *g__Bacillus* |
| OTU1151 | 1.76246146 | *g__Parabacteroides* |
| OTU1020 | 1.733813091 | *g__Zoogloea* |
| OTU1154 | 1.70494101 | *g__norank_f__Obscuribacteraceae* |
| OTU942 | 1.702347453 | *g__norank_f__Muribaculaceae* |
| OTU833 | 1.649877633 | *g__norank_f__Muribaculaceae* |
| OTU1234 | 1.64786381 | *g__Butyricimonas* |
| OTU820 | 1.646830892 | *g__unclassified_o__Bacillales* |
| OTU879 | 1.638596576 | *g__Rheinheimera* |
| OTU1148 | 1.637365307 | *g__Parvibacter* |
| OTU824 | 1.608645573 | *g__A2* |
| OTU886 | 1.606622241 | *g__Acinetobacter* |
| OTU1210 | 1.59269574 | *g__Parabacteroides* |
| OTU1156 | 1.550816424 | *g__Muribaculum* |
| OTU913 | 1.537001918 | *g__norank_f__Muribaculaceae* |
| OTU290 | 1.479912574 | *g__Pantoea* |
| OTU947 | 1.428115018 | *g__norank_f__Muribaculaceae* |
| OTU922 | 1.418585649 | *g__Acinetobacter* |
| OTU971 | 1.417050503 | *g__Cupriavidus* |
| OTU905 | 1.415707668 | *g__Psychrobacillus* |
| OTU144 | 1.413899397 | *g__Sphingomonas* |
| OTU1087 | 1.412859872 | *g__Caulobacter* |
| OTU787 | 1.412136112 | *g__norank_f__Kineosporiaceae* |
| OTU1013 | 1.408348959 | *g__Cupriavidus* |
| OTU502 | 1.3932675 | *g__Cystobacter* |
| OTU1130 | 1.374578563 | *g__Clostridium_sensu_stricto_1* |
| OTU1190 | 1.366929044 | *g__Pseudanabaena_PCC-7429* |
| OTU756 | 1.339339114 | *g__Klenkia* |
| OTU825 | 1.329360598 | *g__Acinetobacter* |
| OTU75 | 1.312864278 | *g__Corynebacterium* |
| OTU815 | 1.306951705 | *g__Cutibacterium* |
| OTU1018 | 1.291019078 | *g__Delftia* |
| OTU1177 | 1.281428791 | *g__Paenibacillus* |
| OTU373 | 1.280171159 | *g__Marmoricola* |
| OTU32 | 1.235621338 | *g__unclassified_k__norank_d__Bacteria* |
| OTU1226 | 1.215657754 | *g__Bacillus* |
| OTU1230 | 1.212139338 | *g__Bacillus* |
| OTU822 | 1.173071649 | *g__Frondihabitans* |
| OTU1229 | 1.153168269 | *g__unclassified_c__Bacilli* |
| OTU584 | 1.144790857 | *g__Streptococcus* |
| OTU793 | 1.126470233 | *g__Knoellia* |
| OTU1141 | 1.117562748 | *g__Alistipes* |
| OTU876 | 1.029802899 | *g__unclassified_k__norank_d__Bacteria* |
| OTU832 | 1.023550386 | *g__Bacillus* |
| OTU346 | 1.016270403 | *g__Lactobacillus* |
| OTU868 | 1.014820265 | *g__Bacillus* |
| OTU725 | 1.001001503 | *g__1174-901-12* |
| OTU1187 | 1.001001503 | *g__Chryseobacterium* |
| OTU594 | 1.001001503 | *g__Adhaeribacter* |
| OTU752 | 1.001001503 | *g__Nocardioides* |
| OTU763 | 1.001001503 | *g__Pseudomonas* |
| OTU776 | 1.001001503 | *g__unclassified_f__Beijerinckiaceae* |
| OTU513 | 1.001001503 | *g__Hymenobacter* |
| OTU632 | 1.001001503 | *g__Terrisporobacter* |
| OTU648 | 1.001001503 | *g__Pseudomonas* |
| OTU848 | 1.001001503 | *g__Nodosilinea_PCC-7104* |
| OTU974 | 1.001001503 | *g__Azospira* |
| OTU1201 | 1.001001503 | *g__unclassified_f__Planococcaceae* |
| OTU586 | 1.001001503 | *g__Endobacter* |
| OTU767 | 1.001001503 | *g__Actinomyces* |
| OTU866 | 1.001001503 | *g__Cyanobium_PCC-6307* |
| OTU973 | 1.001001503 | *g__unclassified_f__Enterobacteriaceae* |
| OTU356 | 1.001001503 | *g__hgcI_clade* |
| OTU755 | 1.001001503 | *g__unclassified_f__Micrococcaceae* |
| OTU1084 | 1.001001503 | *g__Variovorax* |
| OTU975 | 1.001001503 | *g__Pseudomonas* |
| OTU995 | 1.001001503 | *g__Sphingobium* |
| OTU1016 | 1.001001503 | *g__Deinococcus* |
| OTU1159 | 1.001001503 | *g__Paenibacillus* |
| OTU561 | 1.001001503 | *g__Quadrisphaera* |
| OTU1050 | 1.001001503 | *g__Saccharibacillus* |
| OTU870 | 1.001001503 | *g__unclassified_f__Rhizobiaceae* |
| OTU585 | 1.001001503 | *g__Solirubrobacter* |
| OTU378 | 1.001001503 | *g__Neisseria* |
| OTU882 | 1.001001503 | *g__norank_f__Chroococcidiopsaceae* |
| OTU463 | 1.001001503 | *g__Devosia* |
| OTU292 | 1.001001503 | *g__Cetobacterium* |
| OTU83 | 1.001001503 | *g__Tychonema_CCAP_1459-11B* |
| OTU941 | 1.001001503 | *g__Odoribacter* |
| OTU429 | 1.001001503 | *g__Candidatus_Alysiosphaera* |
| OTU718 | 1.001001503 | *g__Altererythrobacter* |
| OTU14 | 1.001001503 | *g__Mucispirillum* |
| OTU424 | 1.001001503 | *g__Skermanella* |
| OTU7 | 1.001001503 | *g__Stenotrophomonas* |
| OTU1032 | 1.001001503 | *g__Luteitalea* |
| OTU33 | 1.001001503 | *g__Bacillus* |
| OTU1183 | 1.001001503 | *g__norank_f__norank_o__Chloroplast* |
| OTU1181 | 1.001001503 | *g__norank_f__norank_o__Chloroplast* |
| OTU736 | 1.001001503 | *g__Hymenobacter* |
| OTU1120 | 1.001001503 | *g__norank_f__norank_o__Chloroplast* |
| OTU156 | 1.001001503 | *g__Clostridium_sensu_stricto_1* |
| OTU662 | 1.001001503 | *g__Adhaeribacter* |
| OTU188 | 1.001001503 | *g__Rhodoplanes* |
| OTU855 | 1.001001503 | *g__CL500-29_marine_group* |
| OTU1225 | 1.001001503 | *g__norank_f__Muribaculaceae* |
| OTU782 | 1.001001503 | *g__unclassified_f__Nocardioidaceae* |
| OTU728 | 1.001001503 | *g__Sphingoaurantiacus* |
| OTU267 | 1.001001503 | *g__Hymenobacter* |
| OTU13 | 1.001001503 | *g__Intestinimonas* |
| OTU802 | 1.001001503 | *g__Aureimonas* |
| OTU991 | 1.001001503 | *g__Rubritepida* |
| OTU699 | 1.001001503 | *g__norank_f__JG30-KF-CM45* |
| OTU573 | 1.001001503 | *g__Terriglobus* |
| OTU524 | 1.001001503 | *g__Ornithinimicrobium* |
| OTU350 | 1.001001503 | *g__Staphylococcus* |
| OTU339 | 0.973189258 | *g__Enterorhabdus* |
| OTU904 | 0.953360592 | *g__unclassified_c__Bacilli* |
| OTU4 | 0.9452795 | *g__norank_f__Desulfovibrionaceae* |
| OTU591 | 0.908584659 | *g__Pseudomonas* |
| OTU1203 | 0.902574627 | *g__unclassified_o__Bacillales* |
| OTU443 | 0.896580734 | *g__Romboutsia* |
| OTU818 | 0.888105067 | *g__Lysinibacillus* |
| OTU20 | 0.887413 | *g__Bacteroides* |
| OTU38 | 0.863304884 | *g__Erysipelatoclostridium* |
| OTU519 | 0.859211431 | *g__Methylobacterium-Methylorubrum* |
| OTU840 | 0.821063923 | *g__Bacillus* |
| OTU471 | 0.818090948 | *g__Methylobacterium-Methylorubrum* |
| OTU878 | 0.792665283 | *g__Cloacibacterium* |
| OTU15 | 0.7830342 | *g__Blautia* |
| OTU415 | 0.776386106 | *g__Lactobacillus* |
| OTU1063 | 0.771259706 | *g__Methylobacterium-Methylorubrum* |
| OTU2 | 0.756405463 | *g__Burkholderia-Caballeronia-Paraburkholderia* |
| OTU16 | 0.746955422 | *g__Sphingomonas* |
| OTU146 | 0.711785631 | *g__Dorea* |
| OTU757 | 0.68486883 | *g__Blastococcus* |
| OTU1195 | 0.669245422 | *g__norank_f__UCG-010* |
| OTU46 | 0.627221653 | *g__Allorhizobium-Neorhizobium-Pararhizobium-Rhizobium* |
| OTU1213 | 0.616471793 | *g__norank_f__norank_o__Clostridia_UCG-014* |
| OTU472 | 0.615320857 | *g__unclassified_f__Acetobacteraceae* |
| OTU1202 | 0.604097597 | *g__Enterorhabdus* |
| OTU1218 | 0.585996855 | *g__Bacillus* |
| OTU976 | 0.577923059 | *g__Pseudomonas* |
| OTU416 | 0.56610102 | *g__Akkermansia* |
| OTU49 | 0.559768989 | *g__norank_f__Eubacterium_coprostanoligenes_group* |
| OTU10 | 0.557258984 | *g__Stenotrophomonas* |
| OTU647 | 0.556730047 | *g__Turicibacter* |
| OTU860 | 0.480327261 | *g__Solibacillus* |
| OTU892 | 0.471406432 | *g__Paenibacillus* |
| OTU1149 | 0.444024778 | *g__unclassified_f__Lachnospiraceae* |
| OTU1165 | 0.429042629 | *g__Nesterenkonia* |
| OTU1139 | 0.399106999 | *g__Marmoricola* |
| OTU1212 | 0.384746084 | *g__unclassified_c__Bacilli* |
| OTU956 | 0.318031526 | *g__Nakamurella* |
| OTU1135 | 0.309418629 | *g__Bosea* |
| OTU758 | 0.285207684 | *g__Sphingomonas* |
| OTU805 | 0.277371435 | *g__Asticcacaulis* |
| OTU481 | 0.2719743 | *g__Rubellimicrobium* |
| OTU1040 | 0.185213689 | *g__Paracoccus* |
| OTU571 | 0.117533005 | *g__Bacillus* |
| OTU919 | 0.108466505 | *g__Clostridium_sensu_stricto_1* |
| OTU940 | 0.100759567 | *g__Anaerotruncus* |
| OTU807 | 0.100759567 | *g__Chroococcidiopsis_SAG_2023* |
| OTU1220 | 0.078218302 | *g__Bacillus* |
| OTU921 | 0.061429743 | *g__Deinococcus* |
| OTU1198 | 0.056523522 | *g__norank_f__Muribaculaceae* |
| OTU961 | 0.049732826 | *g__Paenibacillus* |
| OTU910 | 0.041840754 | *g__unclassified_c__Bacilli* |
| OTU35 | 0.016833495 | *g__Pantoea* |
